# Conversational Agents as Mediating Social Actors in Chronic Disease Management Involving Health Care Professionals, Patients, and Family Members: Multisite Single-Arm Feasibility Study

**DOI:** 10.2196/25060

**Published:** 2021-02-17

**Authors:** Tobias Kowatsch, Theresa Schachner, Samira Harperink, Filipe Barata, Ullrich Dittler, Grace Xiao, Catherine Stanger, Florian v Wangenheim, Elgar Fleisch, Helmut Oswald, Alexander Möller

**Affiliations:** 1 Centre for Digital Health Interventions Department of Management, Technology, and Economics ETH Zurich Zurich Switzerland; 2 Future Health Technologies Programme Campus for Research Excellence and Technological Enterprise Singapore-ETH Centre Singapore Singapore; 3 Centre for Digital Health Interventions Institute of Technology Management University of St Gallen St Gallen Switzerland; 4 Fakultät Digitale Medien Campus Furtwangen Hochschule Furtwangen University Furtwangen Germany; 5 Johns Hopkins University School of Medicine Johns Hopkins University Baltimore, MD United States; 6 Center for Technology and Behavioral Health Geisel School of Medicine Dartmouth College Hanover, NH United States; 7 Department of Child and Adolescent Health Cantonal Hospital Winterthur Winterthur Switzerland; 8 Division of Respiratory Medicine and Childhood Research Center University Children’s Hospital Zurich Zurich Switzerland

**Keywords:** digital health intervention, intervention design, mHealth, eHealth, chatbot, conversational agent, chronic diseases, asthma, feasibility study

## Abstract

**Background:**

Successful management of chronic diseases requires a trustful collaboration between health care professionals, patients, and family members. Scalable conversational agents, designed to assist health care professionals, may play a significant role in supporting this collaboration in a scalable way by reaching out to the everyday lives of patients and their family members. However, to date, it remains unclear whether conversational agents, in such a role, would be accepted and whether they can support this multistakeholder collaboration.

**Objective:**

With asthma in children representing a relevant target of chronic disease management, this study had the following objectives: (1) to describe the design of MAX, a conversational agent–delivered asthma intervention that supports health care professionals targeting child-parent teams in their everyday lives; and (2) to assess the (a) reach of MAX, (b) conversational agent–patient working alliance, (c) acceptance of MAX, (d) intervention completion rate, (e) cognitive and behavioral outcomes, and (f) human effort and responsiveness of health care professionals in primary and secondary care settings.

**Methods:**

MAX was designed to increase cognitive skills (ie, knowledge about asthma) and behavioral skills (ie, inhalation technique) in 10-15-year-olds with asthma, and enables support by a health professional and a family member. To this end, three design goals guided the development: (1) to build a conversational agent–patient working alliance; (2) to offer hybrid (human- and conversational agent–supported) ubiquitous coaching; and (3) to provide an intervention with high experiential value. An interdisciplinary team of computer scientists, asthma experts, and young patients with their parents developed the intervention collaboratively. The conversational agent communicates with health care professionals via email, with patients via a mobile chat app, and with a family member via SMS text messaging. A single-arm feasibility study in primary and secondary care settings was performed to assess MAX.

**Results:**

Results indicated an overall positive evaluation of MAX with respect to its reach (49.5%, 49/99 of recruited and eligible patient-family member teams participated), a strong patient-conversational agent working alliance, and high acceptance by all relevant stakeholders. Moreover, MAX led to improved cognitive and behavioral skills and an intervention completion rate of 75.5%. Family members supported the patients in 269 out of 275 (97.8%) coaching sessions. Most of the conversational turns (99.5%) were conducted between patients and the conversational agent as opposed to between patients and health care professionals, thus indicating the scalability of MAX. In addition, it took health care professionals less than 4 minutes to assess the inhalation technique and 3 days to deliver related feedback to the patients. Several suggestions for improvement were made.

**Conclusions:**

This study provides the first evidence that conversational agents, designed as mediating social actors involving health care professionals, patients, and family members, are not only accepted in such a “team player” role but also show potential to improve health-relevant outcomes in chronic disease management.

## Introduction 

Chronic conditions present a significant risk to the global population, and cause substantial financial and health-related burdens, resulting in low quality of life of those affected [1]. Chronic diseases affected more than half the population of the United States in 2016, representing a leading cause of death, and their prevalence is expected to rise even further [1]. In addition to ongoing treatment and medical oversight, disease management is a key pillar for chronic condition alleviation by aiming to minimize their symptoms, resulting functional impairments, and related exacerbating risks [2].

Successful disease management often requires a trustful collaboration among health care professionals, patients, and their families [3]. In addition, patients require specialized cognitive and behavioral skills to deal with their condition [4,5]. This is especially important for affected children who have to deal with their disease for their upcoming future [3,6].

Digital health interventions are emerging tools for the management of chronic diseases, as they can educate and engage patients through a direct channel that supports communication with physicians and health care professionals [7,8], and enable the scale up of personalized and behavioral interventions at low cost [1,9]. Digital health interventions offer medical care outside the clinical setting to provide ongoing support and communication in everyday monitoring and management [1]. Indeed, several recent studies have provided evidence supporting the patient benefits of such digital interventions, particularly in children and adolescents [10-14]. In addition, conversational agents (ie, computer programs that imitate interaction with human beings) have shown promising results with respect to patient satisfaction [15], therapeutic alliance [16,17], and treatment success [18]. Digital health interventions in the form of mobile apps can be particularly effective for children as they provide an attractive channel for education and management through the possible integration of multimedia content such as audio or video [19]. Conversational agents, as part of such interventions, can act as mediating social actors; that is, they take over not only a significant amount of the intervention delivery in a scalable manner but also coordinate the communication among health care professionals, family members, and patients if required.

This study focused on asthma as a representative chronic condition. Affecting approximately 235 million people, asthma is one of the most common chronic diseases worldwide [20]. Asthma is characterized by reversible airway obstruction [21]. Its symptoms include wheezing, shortness of breath, and coughing [22]. Asthma is associated with high financial and health costs, with total annual asthma costs in the United States estimated at US $56 billion in 2011 [23]. Depending on the country, the mean cost of asthma care per patient per year can range from US $1900 in Europe to US $3100 in the United States [23]. Even though lack of medical treatment leads to significantly reduced quality of life, the management of asthma still presents a daunting challenge because the exact cause is not well known and its appearance varies significantly between individuals [24].

For asthma, specific cognitive skills required for disease management include knowledge about asthma triggers and the importance of medication inhalation adherence, as well as behavioral skills such as the application of correct inhalation techniques. Further, asthma education and health literacy are fundamental to self-management since better understanding of the condition would help patients avoid the negative effects of poor asthma control [25-27]. Low levels of health literacy have been linked with adverse health outcomes, including more frequent hospitalization and longer stays, even after controlling for severity of illness and socioeconomic variables [28,29].

However, young patients still face problems related to both cognitive and behavioral skills that hinder their ability to effectively administer asthma medications [30-35]. For example, knowledge about asthma or important facets of asthma control such as the importance of medication adherence might change over time, making it necessary for patients to continuously update their knowledge base [36-40]. Another common concern is poor technique during medication inhalation, leading to reduced dispersion of the drug in the lungs, and subsequent decreased asthma stability and lowered clinical effectiveness of the delivered drug [41-44].

Numerous mobile apps have been developed for the management of asthma with particular focus on tracking symptoms or medications [45]. Asthma apps targeted at children often include a gamification component to increase engagement, and to familiarize them with aspects of asthma monitoring and management such as medication intake [46,47]. However, and in addition to shortcomings of asthma management related to cognitive and behavioral skills, children often face problems with such technological solutions when they are not integrated into existing health care systems and do not allow for explicit support by health care professionals or family members. Without a dedicated party or mediator, it often becomes a challenge to integrate all of these relevant stakeholders (ie, health care professionals, family members, and the patients themselves) into the disease management process.

Additionally, due to absent or insufficient motivation strategies such as interactivity, proper incentives and rewards [48], and experiential value [49-51], the effects of the previously reported digital interventions in asthma, such as the health condition of the young patients, are prone to be negatively affected by the temporal decline in the patients’ engagement and motivation [10,52-54]. A patient’s motivation to comply with digital interventions and adhere to therapeutic tasks may be further diminished by various factors such as family routines; child-raising issues; social issues [55]; and trust, communication, and empathy with health care professionals [56]. Moreover, there is evidence that shared decision making and collaboration among patients, parents, and health care professionals are key success factors in guided asthma self-management programs with improved adherence and health outcomes [55].

Against this background, our research questions are (1) whether conversational agents would be generally adopted for developing a trustful collaboration among health care professionals, young patients, and their family; and (2) whether they could have a positive impact on the management of asthma in children. To answer these questions, this study had the following objectives: (1) to describe the design of MAX, a conversational agent–delivered asthma intervention that supports health care professionals targeting children-parent teams in their everyday lives; and (2) to assess the (a) reach of MAX, (b) conversational agent–patient working alliance, (c) acceptance of MAX, (d) intervention completion rate, (e) cognitive and behavioral outcomes, and (f) human effort and responsiveness of health care professionals in both primary and secondary care settings.

## Methods

### Design

MAX was designed for the German-speaking part of Switzerland, and was evaluated in two home care settings and four secondary care settings at hospitals. The following subsections describe the design and evaluation procedures in detail.

### Conceptual Model

**Figure 1:**
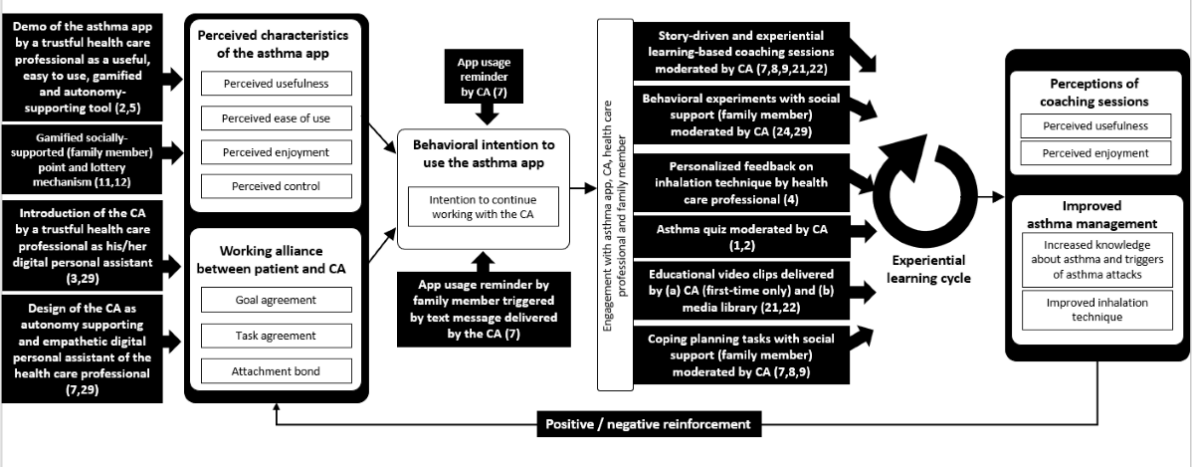
Conceptual model of the intervention. Intervention components are represented by black boxes; behavioral change technique numbers [65] are listed in brackets for each intervention component. CA: conversational agent.

**Figure 2:**
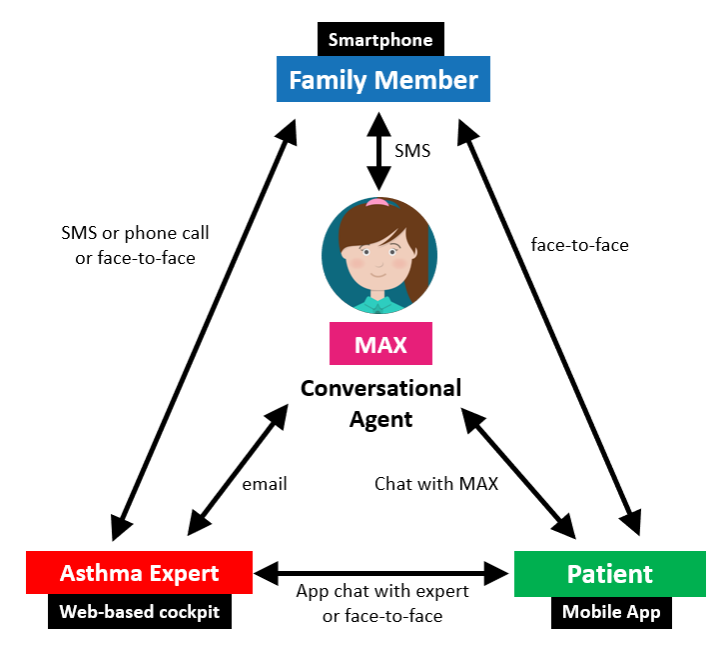
Communication concept of social actors; MAX combines different communication channels and incorporates family members, patients, and asthma experts into on-site and remote counseling sessions.

Following the preparation phase of the multiphase optimization strategy for behavioral interventions [57], we started with the design of the conceptual model of MAX (see Figure 1). The design of the conceptual model was theoretically informed by related work covering asthma management in children (see Introduction), information systems and technology acceptance research [49-51,58], working alliance [59,60] linked to conversational agents [16,61-64], behavior change techniques (BCTs) [65], and experiential learning theory [66]. Moreover, feedback from four asthma experts of the Swiss Lung Association; two pediatric pneumologists of Swiss children’s hospitals; young asthma patients and their parents; and lessons learnt from prior work, in which we developed conversational agents for children with obesity [67,68], were used in the design process. The resulting conceptual model reflects the causal chain triggered by intervention components that target (1) the engagement of young patients with the asthma app, conversational agent, health care professional, and supporting family member (left part of Figure 1), and (2) the outcomes of the intervention (right part of Figure 1).

### Communication Concept

The communication concept of the intervention allowed patients to engage with the asthma app, the conversational agent MAX, health care professionals, and family members via different communication channels. The communication concept is depicted in Figure 2. In line with self-determination theory [69], which describes autonomy (ie, the need to self-regulate one’s experiences and actions as important predictors of engagement [70]), the setup of this communication system allowed patients to independently decide with whom to interact and when, to establish relatedness to all involved stakeholders, and to ultimately increase their competence in the form of improved asthma management.

The conversational agent itself followed a predefined intervention schedule route (see Multimedia Appendix 1 and 2) to communicate with all participating groups (ie, with health care professionals via email, with patients via a mobile chat app, and with a family member via SMS text messaging; see Figure 2). On top of these channels, there was an on-demand option to communicate via these and the other channels (eg, phone call or face-to-face interaction when required or triggered by parents, the patient, or the health care professional).

Besides the mobile app, the intervention offered a web-based MAX interface for health care professionals (see Multimedia Appendix 3), which was only accessible to the participating health care professionals to interact with their patients when required for a coaching session, monitoring their performance, or accessing their personal information such as patient ID. Patients first accessed the MAX app via a QR code printed on a physical card, which was handed out to them by their health care professional at the beginning of the intervention (see Multimedia Appendix 4). Each participant was linked to a personalized code printed on this card. This connection between the patient and health care professional allowed the conversational agent to recognize the treating health professional so that it can link back to its assigned human as needed.

### Intervention Components Triggering Adoption of and Adherence to MAX

To trigger engagement and in line with the theories of planned behavior [71], self-determination theory [69], and technology acceptance research [49,50,58], perceived characteristics of the asthma app (ie, perceived usefulness, ease of use, enjoyment, and control) and working alliance with the conversational agent (ie, goal agreement, task agreement, and attachment bond) were hypothesized to positively influence the behavioral intention to continue working with the conversational agent.

To positively influence the perceived characteristics of the asthma app, health care professionals, who aim to build a trustful relationship with their patients, as this is central in health care situations [72,73], were asked to *demonstrate the app to their patients as a useful, easy to use, gamified, and autonomy-supporting tool* for their asthma management. Toward this end, they provided information on the consequences of behavior to the individual (BCT 2 [65]) and aimed at goal setting (BCT 5 [65]) early on. They introduced patients to the overall communication concept of MAX (see Figure 2), which allowed the integration of all involved stakeholders and the realization of a hybrid ubiquitous coaching approach via on-site and remote counseling sessions. This onboarding activity with health care professionals further aimed at attaining the perception of the conversational agent as a trustworthy social actor that complements the health care professional and family team.

Moreover, a *gamified and socially supported point and lottery mechanism* was implemented as an intervention component to positively influence the patient’s perceived enjoyment of the app, which was designed to influence the subsequent behavioral intention of patients to use the asthma app. This intervention component was informed by BCTs 11 and 12 (prompt review of outcome goals and prompt rewards contingent on effort or progress toward behavior, respectively [65]). In detail, patients received 10 points for their first conversational turns with MAX, the participation in a health literacy quiz, and then for each finished coaching session. An additional 10 points were rewarded for each of the seven sessions a family member was involved in and to support the patient. Upon finishing the intervention within 30 days, points accumulated up to that point were doubled. To this end, patients received automatic reminders about how many days they had left for qualifying to double their points during the program. Another 100 points were awarded when the family member completed a final survey at the end of the intervention and handed out the generated code to the patient to withdraw the bonus points. The final points were converted into chances for a lottery. Three winners were drawn from each participating Swiss canton (for more details on the study design see the Study Design section)*.* Each winner received a gift voucher worth US $50 for the Apple App Store, Google Play Store, or a visit to a local movie theatre.

To build up a working alliance, health care professionals were asked to *introduce the conversational agent as their personal digital assistant* (BCTs 3 and 29, provide information about others’ approval and plan social support/social change, respectively [65]). In addition, we designed the conversational agent *as an autonomy-supporting* (eg, patients were able to control, and set up a date and time of the digital coaching sessions) *and empathetic digital assistant of the health care professional* (eg, the conversational agent introduced itself as the personal assistant of a health care professional by mentioning his/her name, and, several times during the intervention, the conversational agent asked the patients about their emotional state and provided personalized feedback based on their answers) in accordance with BCTs 7 and 29 (action planning and plan social support/social change, respectively [65]).

Moreover, *app usage reminders* were triggered by the conversational agent as in-app notifications (after 1 hour, 1 day, and 3 days of no interaction) and through a separate communication channel such as via SMS text messaging (after 5 days to the patient and after 7 days to the family member’s smartphone) to positively influence the intention of the patient to continue working with the conversational agent. These reminders endorsed action planning (BCT 7 [65]) and further supported the development of relatedness [69] between patients and their parents as important participants of the intervention.

### Intervention Components Triggering Experiential Learning and Outcomes

Four distinct intervention components as depicted in Figure 1 enabled an experiential learning cycle [66], and were assumed to influence the outcomes of the intervention, including perception of the coaching sessions (ie, perceived usefulness and perceived enjoyment) and the improvement of individual asthma management (ie, increased knowledge about asthma and triggers of asthma attacks, and improved inhalation technique). Experiential learning describes learning as a process that is continuously grounded in experience and understood as a holistic process that fosters adaptions of the learner to the surrounding reality [66]. The four cyclic steps that describe this process—active experimentation, concrete experience, reflective observation, and abstract conceptualization [66]—are triggered by the intervention components.

The story-driven and experiential learning–based coaching sessions moderated by the conversational agent as the overarching intervention component foster active experimentation [66], concrete experiences [66], and implement several BCTs (7-9, 21, 22; see [65] for a detailed description and Multimedia Appendix 1 and 2 for an overview of the coaching sessions). For patients, coaching sessions were moderated by the conversational agent MAX, which offered a relatively simple chat-based interface with predefined answer options to multiple-choice questions, free-text input (eg, asking for the participant’s nickname) or number input fields (eg, asking about the participant’s age), and a linguistic style that evokes interpersonal closeness as this is assumed to be positively related to the attachment bond between the patient and conversational agent [59,74]. MAX mimicked the behavior of a real human being chatting by using emojis and some humor to build up a social relationship [75] and working alliance [61] when conversing with patients (see Multimedia Appendix 5 and Multimedia Appendix 6). To address participants’ accountability, MAX referred to earlier tasks and activities, and gave positive reinforcement. The conversational agent could also send out personalized messages every other day to initiate a conversation, in which the dialogue began with a warm greeting, followed by questions about the participants’ mood such as “How are you today?”

In total, the intervention consisted of 14 individual coaching sessions, in which the topics were designed to increase cognitive skills (ie, knowledge about asthma) and behavioral skills (ie, inhalation technique)*.* Patients could perform a maximum of one coaching session per day to reduce smartphone addiction [76], where each coaching session was designed to last between 10 to 15 minutes. Several coaching sessions required the aid of the supporting family member, such as to film the patient performing an inhalation (see Multimedia Appendix 7 for a representative video clip). The family member was invited by the MAX conversational agent via a corresponding SMS text message at the time the patient made the appointment for that specific coaching session. A detailed schedule of the intervention with an overview of the coaching sessions is outlined in Multimedia Appendix 1.

Assuming that the need to self-regulate one’s experiences and actions (as an important predictor of engagement [70], as posited by self-determination theory) is also true for digital interventions, the intervention schedule was flexible, which is an innovative approach compared to other interventions [17,68,77-79], and enabled accommodation to the patients’ specific needs such as school stress or sickness. Patients could individualize their intervention schedule since they had the option to postpone exercises at their own discretion. This gave patients significant control over the interaction progress and its overall duration. In theory, they could prolong their intervention substantially, but the above-described points reward system incentivized the completion of the program within 30 days by doubling all achieved points when patients complied to this time frame.

The curriculum and storytelling aspects of the intervention were derived from a validated Swiss health literacy comic for children with asthma published by the Swiss Lung Association [80]. Based on this comic, one expert in digital media, didactics, and learning theories wrote a digital health literacy storybook (see Multimedia Appendix 8 and Multimedia Appendix 9), including scripts for 11 health literacy video clips for children with asthma. The storybook was proofread and validated by two asthma experts from the Swiss Lung Association and two pediatric pneumologists. Additionally, established video clips covering correct inhalation techniques for children with asthma were integrated into intervention coaching sessions. These video clips had been produced under the direction of Swiss health care professionals, and are currently used by several Swiss hospitals and patient organizations in their health literacy programs (for links to the video clips see Multimedia Appendix 1 and 2).

Concrete learning experiences [66] were enabled through the intervention component of *behavioral experiments with* *social support (family member) moderated by the conversational agent*. This design allowed patients to relate to the conversational agent and to their social support person. The behavioral experiments addressed asthma management and aimed at improving patients’ competence with asthma management [69]. In addition, they enabled environmental restructuring (BCT 24 [65]) and planning of social support/social change (BCT 29 [65]).

**Figure 3:**
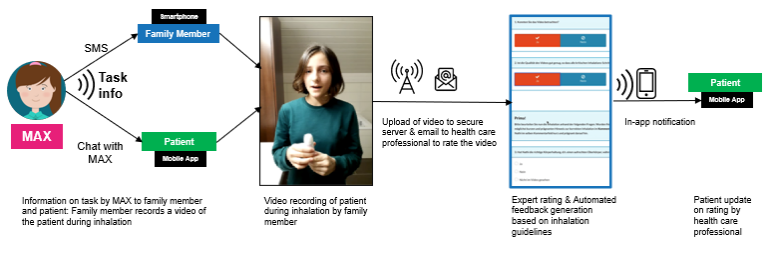
The MAX intervention integrates patients, family members, and health care professionals, and allows a ubiquitous experiential learning experience. An example of session 1 is shown here, in which patients were prompted by the MAX conversational agent to record a video of themselves during inhalation with the help of their support family member, who were additionally informed about the task via SMS text message. Once patients had created and uploaded the video to a secure server, health care professionals received an email to assess the video clip with regard to inhalation mistakes. Patients received their final feedback with comments via in-app notification. See Multimedia Appendix 7 for a representative video clip.

During onboarding, health care professionals checked the inclusion criteria with the help of a study recruitment assessment sheet (see Multimedia Appendix 10 and Multimedia Appendix 11) when patients were interested in participating. When patients decided not to participate in the study, health care professionals noted down the corresponding reasons. Further, patients chose their supporting family member and provided their own and their parent’s mobile phone numbers to enable communication via the asthma app and mobile phone. Family members provided support to young patients as the intervention component. For example, they were asked to record a short video clip during inhalation as part of a coaching session or filled out a final intervention survey that enabled the young patients to gain more points for the above-described lottery. Figure 3 depicts a representative workflow of the integration of the different stakeholders into the MAX intervention in the course of a behavioral intervention with social support (see Multimedia Appendix 7 for a representative video clip). The family member and the patient are notified over their respective communication channels (ie, SMS and in-app) about an upcoming task. Upon completing the task (in this example, recording the patient during inhalation to evaluate any inhalation mistakes), the conversational agent MAX uploads the video on a secure server and triggers an email notification to the child’s health care professional to review the video. The health care professional then assesses the inhalation according to predefined inhalation guidelines (eg, “Did [patient name] exhale enough before inhalation?”) with the tags “correct,” “not correct,” or “not visible in the video.” According to these assessments, an automated feedback message is generated, which could be personalized by the health care professional. In a last step, the health care professional sends the personalized feedback message via the web-based MAX interface for health care professionals and the patient receives it as an in-app notification in a separate “health care professional” chat channel. Depending on the severity of the inhalation mistakes, as indicated by the health care professional with an additional yes/no tag, the MAX conversational agent would ask the patient and supporting family member to redo the video recording of the inhalation technique at the beginning of the next coaching session. The goal of this session is to improve the current workflow by extending the reach of health care professionals into the everyday lives of patients in an efficient way without compromising the quality of care and working alliance. That is, parents do not have to arrange a corresponding on-site consultation and travel to the hospital, while at the same time, a standardized assessment of the inhalation video clips as outlined above may even increase the quality of the feedback, and with it the working alliance between the patient and health care provider.

The intervention components *personalized feedback on inhalation technique by the health care professional, asthma quiz moderated by the conversational agent*, and *educational video clips* *delivered by (a) the conversational agent (first time only) and (b) media library* allowed for reflective observation [66]. In particular, health care professionals assessed the inhalation technique based on video clips recorded by a patient’s family member, provided individual feedback to the patients, and provided normative information about others’ behaviors (BCT 4 [65]). This was done via a dedicated chat channel in the web-based MAX interface for health care professionals and the mobile MAX app (see Figure 3), and during on-site visits. This interaction setup extended the dyadic interaction between the patient and conversational agent, resulting in a ubiquitous experiential learning experience besides fostering the relatedness between patients and health care professionals as relevant interaction partners [69].

Health care quizzes were an integral part of the intervention. Patients took a health care quiz at the beginning and end of the intervention as well as short quizzes that were integrated into the conversational turns with the conversational agent MAX. These elements of gamification aimed to increase cognitive skills and provided information on consequences of behavior, both in general as well as to the individual (BCT 1 and 2 [65]). Participants could choose between multiple answers and received feedback depending on the accuracy of their chosen answer. In line with self-determination theory, the quizzes and educational video clips, which were informed by BCT 21 and 22 (provide instruction on how to perform the behavior and model/demonstrate the behavior, respectively [65]), aimed at strengthening the individual competence of the young patients for managing their health condition [69].

The intervention component *coping planning tasks with social support (family member) moderated by a conversational agent* closed the experiential learning cycle. This component allowed patients to engage in abstract conceptualization [66] of the behavioral and cognitive skills that they had learned previously. In addition, this intervention component supported the improvement of asthma management as the intervention outcome. In line with self-determination theory [69], this further allowed patients to acquire overall increased competence via the integration of BCTs 7-9 (action planning, barrier identification/problem solving, and set graded tasks, respectively [65]).

Finally, we assume that there is a positive/negative reinforcement link that connects the outcomes of the conceptual model with the perceived characteristics of the app and working alliance with the conversational agent. This encourages patients to continue working with the conversational agent and increases engagement behavior, especially for young patients. That is, if neither the coaching sessions are perceived useful and joyful nor improvements of asthma management can be observed as a result of actual participation in the intervention, then engagement in the intervention will likely decrease, which has been shown in related interventions [81,82].

### Technical Implementation

The intervention was developed with the open-source software platform MobileCoach [67,83,84], which has already been used successfully for various clinical and public health interventions [17,68,77-79,85,86] and ecological momentary assessments [87-89]. MobileCoach is available under the academia- and industry-friendly open-source Apache 2.0 license. MobileCoach-based interventions are delivered via SMS text messages, and via mobile apps for the Android and iOS operating systems. Moreover, MobileCoach-based interventions use a conversational agent for intervention delivery. MobileCoach client apps for iOS and Android use in-app encryption of user data, including password-protected access to MobileCoach Designer, a web-based interface for intervention authors, and a web-based MAX interface for health care professionals for chat interactions with human health coaches. Additionally, secure sockets layer (SSL) encoding was implemented to ensure privacy and safety of any data transfer between the mobile apps, the web-based MAX interface for health care professionals, MobileCoach Designer, and the MobileCoach server.

### Costs of Intervention Components

Assessing the cost of each intervention component is relevant for real-life implementations [57]. Therefore, economic factors (eg, budgets of hospitals or health care professionals’ time allocations) also need to be taken into consideration. The development costs of the MAX intervention, which is currently not classified as software related to a medical device in Switzerland as it remains a prototype and not a product, sums up to approximately US $250,000. This includes costs for the storybook, software development, project management, artwork, and production of video clips and the personalized QR code cards. Moreover, other costs per participant are linked to intervention components that involve either incentives (see above) or efforts by health care professionals. Regarding the latter, there are three such intervention components in the MAX intervention. First, health care professionals carry out the onboarding of patients, which encompasses two intervention components: (1) *demo of the asthma app by a trustful health care professional as a useful, easy to use, gamified, and autonomy-supporting tool*; and (2) *introduction of the conversational agent by a trustful health care professional as his/her digital personal assistant* (see Figure 1). Costs associated with these two intervention components are time needed for preparation, performance, and potential postprocessing of this task. Second, providers were involved in the assessment of short video clips sent by the participants as described by the intervention component *personalized feedback on inhalation technique by a health care professional*; see Figure 3 for a detailed explanation and illustration of this task and the according process. Here, associated costs concerned the time needed to assess the video clip and compile related feedback. This process was costly due to the economic costs of health care professionals but could be reduced to a certain extent by automatically providing a video tutorial showing how to perform the inhalation assessment with the web-based MAX interface for health care professionals. Since the video tutorial was integrated into every email that triggered an assessment, access to the tutorial was straightforward and thus of low cost. All other intervention components have low running costs as they are scalable due to their digital setup (eg, interaction over a mobile app and with a conversational agent, digital lottery, coaching sessions moderated by the conversational agent).

### Evaluation of the Intervention

#### Study Design

MAX was assessed in a single-arm feasibility study in two home care settings offered by the Swiss Lung Association and in four secondary care settings at hospitals in the German-speaking part of Switzerland. The study was approved by the institutional review board of ETH Zurich (reference number: EK 2018-N-59).

#### Sample Size Considerations

The primary objective of this single-arm feasibility study was to develop, implement, and test the MAX intervention. Therefore, the study was exploratory by nature and did not include a detailed power analysis to determine a particular sample size. However, to identify a relevant amount of usability problems, at least 20 participants were required according to heuristics in usability engineering [90]. Moreover, to assess the potential reach of the intervention, we decided to approach between 90 and 100 participants.

#### Inclusion Criteria

The following inclusion criteria were defined and outlined in the corresponding intervention flyer (see Multimedia Appendix 12 and Multimedia Appendix 13)*:* (1) 10- to 15-year-old German-speaking patients diagnosed with asthma who have access to a smartphone with Google’s Android (Version 4.1 or higher) or Apple’s iOS (9.3 or higher) operating system and internet access via a data contract (3G/LTE) or wireless LAN (Wi-Fi) to watch the health literacy video clips, to interact with the conversational agent, and to fill out the online surveys; and (2) availability of a German-speaking family member of the patient (usually mother, father, or older sibling) who has access to a smartphone with internet capability via a data contract (3G/LTE) or wireless LAN (Wi-Fi) to be able to receive the SMS text messages from MAX and to fill out the online survey at the end of the intervention. This supporting family member must be motivated to support the young patient every second intervention day. There were no exclusion criteria.

#### Recruitment and Management of Study Participants

The participants were recruited during a 3-month period from January to April 2019 via participating health care professionals at six study sites in Switzerland. The study sites were two home care settings offered by the Swiss Lung Association (one in the canton Bern and one in the canton Thurgau) and four secondary care settings at hospitals in the German-speaking part of Switzerland. The health care professionals received instructions on how to install and use the mobile app before the start of the intervention. Additionally, health care professionals were provided with study instructions so they could consistently recruit and manage their patients during the study (see Multimedia Appendix 14 and Multimedia Appendix 15)*.* This document (and every triggered email when an inhalation video clip was submitted) also included a link to a video tutorial that shows how to perform the inhalation assessment with the web-based MAX interface for health care professionals. They were also trained to introduce the MAX conversational agent as their personal digital assistant. The health care professionals recruited patients with a flyer that was personalized for each health care expert during their consultation hour (see Multimedia Appendix 12 and Multimedia Appendix 13 for examples of a personalized flyer), or via email, post, or telephone. Additionally, participants could access a website [91] for more information on the intervention (eg, with a demonstration video clip showing chatting with the MAX conversational agent), study participation, and frequently asked questions. If a candidate was interested in participating, inclusion criteria were checked by the health care professional, or, if the patient was not interested, corresponding reasons were noted down to better understand the patient’s decisions (see Multimedia Appendix 10 and Multimedia Appendix 11). After reading more detailed study information (see Multimedia Appendix 16 for the German version) and signing the consent form (see Multimedia Appendix 17 for the German version), the health care professionals gave the patients their personal MAX intervention card in the form of a business card with a QR code (see Multimedia Appendix 4). The QR code could be used with the standard photo app of a smartphone (capable of reading QR codes) and automatically forwarded the patient to either the Android or Apple store, depending on the type of their smartphone, to download the mobile app.

#### Measures

To assess the various aspects of the intervention, we used basic demographic, asthma-related, and intervention-related information, including age, gender, years since asthma diagnosis, the supporting family member during the intervention (eg, mother), mobile operating system used, and perceived uncertainty with asthma management. For the latter, this would include the conversation agent stating: “I have been taught some things about asthma by my development team, but I am still unsure from time to time. I'm sure you feel the same?” The answer options are “No, I am an asthma expert” (1), “I know quite well how to manage my asthma” (2), “Every now and then I feel insecure too” (3), and “Yes, I have been uncertain a lot before” (4). In addition, the following metrics and instruments were assessed.

The *reach* of the intervention was measured by the ratio of approached participants to those who started to interact with the conversational agent MAX on the mobile app. Reasons for nonparticipation were also gathered.

*Working alliance* between the patient and the conversational agent MAX was assessed with a German-adapted version of the Session Alliance Inventory [92] after coaching sessions 2, 8, and 14 (eg, “MAX and I respect each other” with answers anchored on a 7-point Likert scale ranging from 1 [“never”] to 8 [“always”]; see Multimedia Appendix 18).

Acceptance of the intervention was assessed in several ways. First, perceived usefulness (“The app helped me to increase my knowledge about my asthma”), ease of use (“The app was easy to use”), enjoyment (“I found the app enjoyable”), control (“I could control many aspects of the app”), and usage intention (“How much would you like to continue working with MAX?”) were assessed by patients at the end of the intervention with instruments adapted from information systems research [50,93]. Single-item measures were used to reduce the burden of the intervention and answers were anchored on 7-point Likert scales ranging from “strongly disagree” (1) to “strongly agree” (7). Second, to obtain a more granular assessment for each coaching session, perceived usefulness (“Did you learn something new?” with answer options “No, I knew everything,” “Yes, some new aspects,” and “Yes, a lot of new aspects”) and perceived enjoyment (“Did you enjoy today’s lesson?” with answer options anchored on a 5-point Likert scale ranging from 1 [“strongly disagree”] to 5 [“strongly agree”]) were assessed at the end of each of the 14 coaching sessions randomly. A random assessment procedure with a maximum chance of 50% was implemented to reduce the burden of the intervention. If a participant had assessed the previous session, no assessment was triggered. Third, participation of the supporting family member (“Have you been supported today by the person you indicated?” with answer options “yes” and “no, unfortunately not”) was measured at the end of each coaching session, which asked for social family support (ie, in sessions 1, 3, 5, 7, 8, 9, and 12). Fourth, during the setup procedure of the mobile app, we measured which of the two gender-specific characters of the MAX conversational agent (either the female *Maxime* or male *Maximilian*) was selected. Fifth, based on app usage data, we measured when participants dropped out of the intervention (ie, did not use it for 60 days). Sixth, we assessed the number of conversational turns between patients, health care professionals, and the conversational agent MAX. Finally, we also collected positive aspects of the intervention (“What did you really like about the intervention?”) and suggestions for improvement (“What needs to be definitely changed in a future version?”) from patients via an in-app conversation with the MAX conversational agent, the supporting family member via a web-based survey for which the MAX conversational agent sent a link by SMS to the family member, and health care professionals via a personal interview conducted by SH. All interview items are available in Multimedia Appendix 17.

Knowledge about asthma (ie, *cognitive skill*) was assessed at the beginning of the intervention (ie, at the end of the introductory chat with the conversational agent MAX) and in the last session by a validated health literacy quiz for children with asthma, with a quiz score ranging from 0 (no knowledge) to 11 (good knowledge) [80,94] (see Multimedia Appendix 19).

The inhalation technique of each patient (ie*, behavioral skill*) was systematically assessed by the patient’s responsible health care professional with the help of predefined evaluation criteria These criteria were developed by health care professionals of the Swiss Lung Association and the participating pediatric pneumologists. The number of mistakes was counted, and it was decided for each assessment and health care professional whether there was a serious, potentially life-threatening, inhalation mistake.

Intervention completion rate was assessed by dividing the number of participants who finished the intervention within 60 days by the number of participants who started to interact with the conversational agent MAX.

We measured *human effort and responsiveness of health care professionals* to better understand the costs per patient related to the intervention. Here, these costs refer to (1) the onboarding time per patient, including a demo of the app and an introduction of the conversational agent MAX such as the time needed for preparation, performance, and potential postprocessing of this task; (2) the assessment of video clips with the time needed to assess the video clip and compile feedback; and (3) the number of conversational turns in the manual/human-managed chat channel of the MAX app. For the first cost aspect, we asked the health care professionals after the intervention to estimate the average onboarding time. For the second cost aspect, we objectively measured the duration required to review the video clips by health care professionals and the technical quality of the video clips (eg, “Did [patient name] exhale enough before inhalation?”). For the third cost aspect, we counted and compared the number of conversational turns between the patient and (a) the MAX conversational agent and (b) the health care professionals to better understand the extent to which the intervention can be delivered in a scalable way. In addition, we measured the number of SMS reminders sent to patients and the supporting family members, since these also trigger costs.

Finally, we measured the time until patients received their feedback (ie, from the moment the video clip was submitted via the mobile app until the feedback was provided) as a further aspect of *human effort and responsiveness of health care professionals*.

## Results

### Participant Characteristics

The descriptive statistics of the study participants are shown in Table 1. Out of the 49 participants who started interacting with MAX, 33 were male with an average of 12 years of age and 5.6 years since receiving the asthma diagnosis. Only 13 of the participants indicated that they were uncertain a lot (n=2) or every now and then (n=11) in managing their asthma. The majority chose their mother as the supporting family member and iOS was used slightly more often than the Android operating system.

**Table 1:** Descriptive statistics of the patient-derived quantitative measures (N=49).

Construct		Respondents, n (%)	Mean (SD)
**Demographic and asthma-related data**			
	Females	16 (33)	N/A^a^
	Males	33 (67)	N/A
	Age	49 (100)	12.04 (1.54)
	Years since asthma diagnosis	39 (80)	5.61 (4.17)
	Perceived uncertainty with asthma (measured in Coaching Session 4)	44 (90)	2.05 (0.81)
**Mobile operating system**			
	Android	22 (45)	N/A
	iOS	27 (55)	N/A
**Supporting family member**			
	Mother	31 (63)	N/A
	Father	9 (18)	N/A
	Older brother	2 (4)	N/A
	Older sister	3 (6)	N/A
	Other	3 (6)	N/A
**Patient-MAX CA^b^** **working alliance**			
	Coaching Session 2	44 (90)	6.34 (0.73)
	Coaching Session 8	39 (80)	6.14 (0.96)
	Coaching Session 14	36 (73)	6.34 (0.87)
**Technology acceptance of mobile app**			
	Perceived usefulness	36 (73)	6.42 (1.09)
	Perceived ease of use	36 (73)	6.75 (0.65)
	Perceived enjoyment	36 (73)	6.47 (1.06)
	Perceived control	36 (73)	5.53 (1.78)
	Intention to continue working with the MAX CA	36 (73)	5.58 (1.73)
**Perceived usefulness of coaching session**			
	Coaching Session 1	22 (45)	1.91 (0.68)
	Coaching Session 2	10 (20)	2.50 (0.53)
	Coaching Session 3	12 (24)	2.58 (0.67)
	Coaching Session 4	14 (29)	2.36 (0.74)
	Coaching Session 5	13 (27)	2.54 (0.78)
	Coaching Session 6	14 (29)	2.29 (0.73)
	Coaching Session 7	13 (27)	2.38 (0.77)
	Coaching Session 8	13 (27)	2.31 (0.77)
	Coaching Session 9	12 (24)	2.58 (0.67)
	Coaching Session 10	16 (33)	2.50 (0.73)
	Coaching Session 11	11 (22)	1.82 (0.75)
	Coaching Session 12	13 (27)	2.38 (0.87)
	Coaching Session 13	16 (33)	1.88 (0.62)
	Coaching Session 14	13 (27)	2.15 (0.80)
	Total	192 (100)	2.28 (0.74)
**Perceived enjoyment of coaching session**			
	Coaching Session 1	22 (45)	4.91 (0.29)
	Coaching Session 2	10 (20)	4.70 (0.48)
	Coaching Session 3	12 (24)	4.83 (0.39)
	Coaching Session 4	12 (24)	4.83 (0.39)
	Coaching Session 5	13 (27)	4.69 (1.11)
	Coaching Session 6	14 (29)	4.79 (0.58)
	Coaching Session 7	13 (27)	5.00 (0.00)
	Coaching Session 8	13 (27)	4.69 (0.48)
	Coaching Session 9	12 (24)	4.83 (0.39)
	Coaching Session 10	16 (33)	4.81 (0.54)
	Coaching Session 11	11 (22)	4.64 (0.92)
	Coaching Session 12	13 (27)	4.69 (0.85)
	Coaching Session 13	16 (33)	4.75 (0.58)
	Coaching Session 14	13 (27)	5.00 (0.00)
	Total	190 (100)	4.81 (0.56)
**Duration to complete the intervention/coaching session^c^**			
	Average duration (days)	37 (76)	21.46 (11.55)
	Average days per coaching session	37 (76)	1.43 (0.77)
**Conversational turns between the patients and the MAX CA**			
	Participants finishing the intervention	37 (76)	365.49 (11.85)
	Participants not finishing the intervention	12 (25)	129.58 (59.86)
**Conversational turns between the patients and health care professionals**			
	Participants finishing the intervention	37 (76)	1.68 (1.68)
	Participants not finishing the intervention	12 (25)	1.00 (1.35)
**In-app (free of cost) and SMS reminders sent to patients and supporting family member**			
	Participants finishing the intervention	37 (76)	11.57 (8.46)
	Participants not finishing the intervention	12 (24)	20.75 (15.88)
**SMS reminders sent to patients after 5 days of nonactivity**			
	Participants finishing the intervention	37 (76)	0.24 (0.86)
	Participants not finishing the intervention	12 (24)	2.50 (1.68)
**SMS reminders sent to supporting family member after 7 days of nonactivity**			
	Participants finishing the intervention	37 (76)	0.14 (0.67)
	Participants not finishing the intervention	12 (24)	2.00 (1.28)
**Asthma knowledge (cognitive skills)**			
	Asthma quiz score onboarding (pretest)	48 (98)	7.73 (2.24)
	Asthma quiz score coaching session 14 (posttest)^d^	48 (98)	8.79 (2.27)
	Asthma quiz score Coaching Session 14 (posttest)^e^	37 (76)	9.43 (1.76)

^a^N/A: not applicable.

^b^CA: conversational agent.

^c^Based on data from participants finishing the intervention.

^d^Last observation carried forward (ie, the pretest value was used for 11 participants).

^e^Complete cases, no missing values.

**Figure 4:**
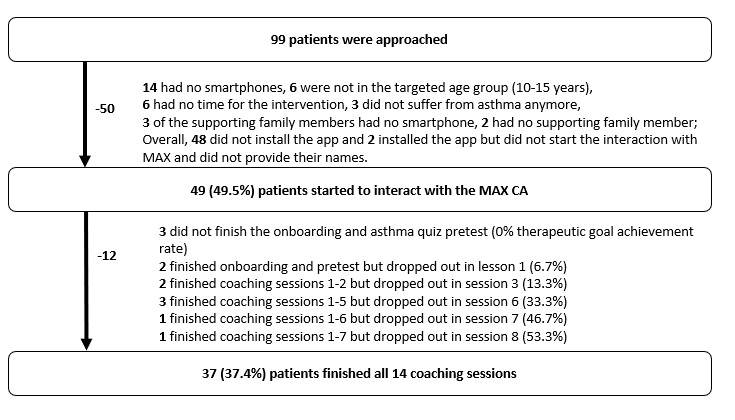
Subject acquisition and participation flow chart. CA: conversational agent.

**Figure 5:**
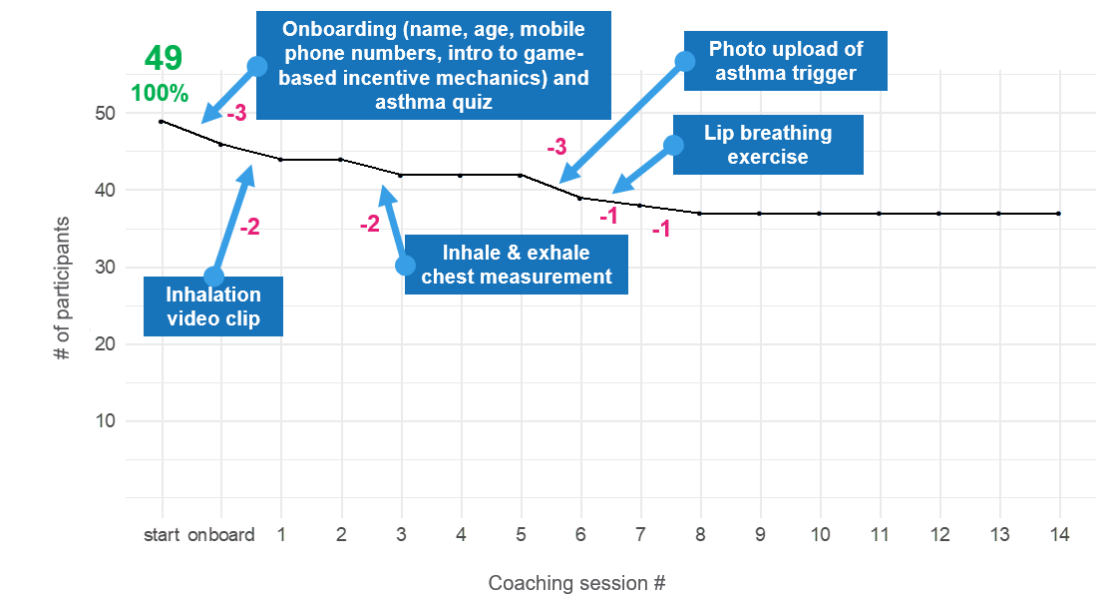
Participants for each coaching session and potential reasons for dropouts.

The flow chart of the MAX intervention, including details for nonparticipation and dropouts, is shown in Figure 4. Reach was 49.5% with 49 out of 99 approached patients downloading the app and starting to interact with the MAX conversational agent. Availability of a smartphone was the major reason for nonparticipation (n=14, 14%), and the most frequent dropouts happened during onboarding (n=3) and Coaching Session 6 (n=3). To better understand sessions after which participants no longer interacted with the MAX conversational agent (ie, they dropped out), Figure 5 indicates the key task involved in each “dropout session.” The effort to complete a specific coaching session and disclosing personal information (eg, recording the inhalation technique with the face of the patient) may have led to dropout. Participants who finished the intervention (n=37) did so on average within 3 weeks, which was within the incentivized duration of 4 weeks.

### Session Alliance Inventory

The session alliance inventory indicated high working alliance ratings between the young patients and the MAX conversational agent from the very beginning of the intervention until the end (Table 1).

### Technology Acceptance

Technology acceptance perceptions of the young patients regarding the mobile app are shown in Table 1. All mean values lie clearly above the neutral scale value of 4, indicating positive evaluations of the mobile app. Moreover, patients learned new aspects about asthma management and enjoyed the coaching sessions. Out of 275 coaching sessions, in which a family member was asked to support the young patients, patients indicated 269 times (97.8%) that they were supported by a family member. For the gender-specific choices of the MAX conversational agent, all participants chose the character corresponding to their own indicated gender.

### Qualitative Feedback

The detailed qualitative feedback with representative quotes is provided in Multimedia Appendix 20. First, patients liked the educational content of the intervention and the text-based features of the conversational agent the most. Second, supporting family members also highlighted the educational content besides the experiential value of the intervention. Third, health care professionals positively emphasized the perceived ease of use and the significant supporting role of family members in this intervention. With respect to improvement suggestions of the intervention, patients indicated that there was too much predefined text. This concern was also shared by supporting family members. Health care professionals indicated that lack of access to smartphones, especially for young patients, was a limiting factor to further increase the reach of the intervention. In addition, health care professionals indicated the following features to be considered in a future version. First, they would prefer an adaptation of the inclusion criteria, especially regarding the age range, to be able to further address younger and older patients. Second, they suggested cooperating with pneumologists and general practitioners to expand the intervention to other health-related topics or diseases (eg, eating disorders or diseases with similar complexity to asthma). Third, they suggested integrating further interaction between the health care professionals and patients (eg, follow-up questions).

### Asthma Knowledge

Asthma knowledge (cognitive skills) scores at the beginning and end of the MAX intervention are shown in Table 1. Paired-sample *t* tests revealed a significant increase in scores and large effects with two approaches: a complete case analysis (n=37, t_36_=–3.68, *P*<.001, *d*=1.19) and with the baseline observation carried forward (n=48, t_47_=–3.54, *P*<.001, *d*=0.91).

### Intervention Completion Rate

The intervention completion rate was 75.5%, as 37 out the 49 patients finished the intervention.

Overall, 42 inhalation video clips were recorded and submitted to the health care professionals (Table 2). All of these clips had sufficient technical quality for evaluation. The majority of inhalant medications used were dry powder inhaler and metered-dose inhaler. The health professionals’ assessments of the inhalation techniques (behavioral skills) based on these video clips are listed in Table 3. In summary, health care professionals identified 0.9 inhalation mistakes in each video clip (N=42). For two video clips, three serious inhalation mistakes were identified, eliciting a feedback to resend a corrected video clip. After resubmission, no severe inhalation mistakes could be identified in the second video clip.

**Table 2:** Descriptive statistics of inhalation video clip assessments (N=42).

Variable		n (%)	Mean (SD)
**Inhalant**			
	Dry powder inhaler (Turbuhaler)	17 (40)	N/A^a^
	Metered-dose inhaler	16 (38)	N/A
	Dry powder inhaler (Diskus)	9 (21)	N/A
**Duration of video clip assessments (seconds)**			
	Primary care providers (N=2)	14 (33)	409.51 (346.48)
	Secondary care providers (N=4)	28 (67)	126.94 (102.80)
	Total	42 (100)	221.13 (251.39)
**Inhalation mistakes identified per submitted video clip**			
	Primary care providers (N=2)	14 (33)	0.93 (0.83)
	Secondary care providers (N=4)	28 (67)	0.93 (1.30)
	Total	42 (100)	0.93 (1.16)
**Days until feedback was provided (including weekends)**			
	Primary care providers (N=2)	14 (33)	2.25 (1.83)
	Secondary care providers (N=4)	28 (67)	2.40 (1.81)
	Total	42 (100)	2.34 (1.80)

^a^N/A: not applicable.

**Table 3:** Inhalation technique assessments by health care professionals.

Assessment question		Yes, n (%)		No, n (%)		Not visible on the video, n (%)	
**Questions for all assessments (N=42)**							
	Has _^a^ the correct posture (ie, an upright upper body) during inhalation?		42 (100)		0 (0)		0 (0)
	Did _ load/prepare the device correctly?		30 (71)		4 (10)		8 (19)
	Did _ exhale enough before inhalation?		30 (71)		8 (19)		4 (10)
	Did _ inhale deeply and long enough through the mouth during inhalation?		34 (81)		7 (17)		1 (2)
	Did _ hold his breath for 5-10 seconds? OR an alternative for the metered-dose inhaler: Were 10 calm breaths taken via the upstream chamber?		38 (90)		4 (9.52%)		0 (0)
	Did _ exhale slowly afterward?		33 (79)		5 (11.90%)		4 (10)
**Additional metered-dose inhaler questions (N=16)**							
	Has the cap of the dosing aerosol been removed?		13 (81)		0 (0)		3 (19)
	Was the metered dose aerosol shaken before inhalation?		10 (63)		2 (13)		4 (25)
	Was the upstream chamber used?		15 (94)		1 (6)		0 (0)
	Was the upstream chamber clean? (N=15^b^)		14 (93)		0 (0)		1 (7)
	Was the age-appropriate upstream chamber used? (mouthpiece, mask) (N=15^b^)		15 (100)		0 (0)		0 (0)
	Was there a whistling sound of the upstream chamber during inhalation? (inhaled too strongly and quickly)^c^ (N=15^b^)		11 (73)		3 (20)		1 (7)
	Did _ trigger the device at the right time during inhalation?		15 (100)		0 (0)		0 (0)
Was exhaled incorrectly into the powder inhaler so that there is a risk of clumping?^c ,d^			22 (85)		3 (12)		1 (4)
Has _ rinsed their mouth with water after inhalation or eaten anything?^e^			7 (22)		2 (6)		23 (72)

^a^_indicates the patient’s name.

^b^Includes only those who answered “yes” to using the upstream chamber.

^c^Reverse coded.

^d^Additional question for those using the dry powder inhaler only (N=26).

^e^Additional question if the inhalant contained cortisol (N=32).

### Human Effort and Responsiveness of Health Care Professionals

For the human effort and responsiveness of health care professionals (ie, to better understand the per-patient costs related to the intervention), the average time of the app onboarding process (excluding study-specific discussions) was approximately 15 minutes. Moreover, it took health care professionals an average of 221 seconds to assess the videos clips, with a clear difference observed between health care settings (average of 410 seconds in the primary care setting and 127 seconds in the secondary care setting; see Table 2). For the responsiveness of health care professionals, patients received feedback on their submitted video clips after an average of approximately 2.4 days (Table 2). In contrast to the assessment time, there were no differences between the health care professionals of the primary and secondary care settings. For the distribution of conversational turns, 99.5% (15,078/15,152) took place between patients and the MAX conversational agent, and only 0.5% (74/15,152) occurred between patients and health care professionals (Table 1). This indicates a very low amount of human effort (ie, 1-1.7 conversational turns between a health care professional and patient; see Table 1). Finally, between 0.1 and 2.5 SMS reminders were sent out on average per patient by the MAX conversational agent (Table 1), in addition to the 7 SMS text messages that were sent out to invite the supporting family members to join the seven “social support” coaching sessions.

The depersonalized data can be found on the Open Science Framework [95] for replication purposes and future analyses. It should be noted that not all data can be published due to ethical considerations and to protect the privacy of the participants of this study.

## Discussion

### Primary Findings

We have described the design of MAX, a smartphone-based and conversational agent–delivered asthma intervention that supports health care professionals targeting children-parent dyads in their everyday lives. Although there have been recent review papers discussing the use of conversational agents in health care [63,96-101], the current conversational agent is the first (to the best of our knowledge) that takes over the role of a scalable social actor framed as a scalable assistant of a health care professional that mediates communication among various relevant stakeholders in the context of chronic disease management. For this purpose, the MAX conversational agent uses several communication channels (eg, in-app chat, email, and SMS), and therefore “lives” not only on a smartphone in the pocket of a patient but is rather omnipresent (ie, MAX appears on the phones of family members, such as via SMS, or on desktop or tablet computers of health care professionals, such as via emails and the web-based MAX interface for health care professionals). This is also the first assessment of this type of mediating conversational agent outside of a lab setting, as many other conversational agents have been assessed [96], but rather in a realistic longitudinal intervention field study in a complex sociotechnical system with various stakeholders. With the MAX conversational agent, we were also able to show how to extend the reach of health care professionals into the everyday lives of patients in an efficient way without compromising the quality of care and working alliance. This is especially relevant in times of social distancing such as during the ongoing COVID-19 pandemic.

The design of MAX was driven by an interdisciplinary effort that resulted in a conceptual model with intervention components informed by human behavior and experiential learning theories [61,65,66], findings from technology acceptance research [49,51,58,102], and prior experiences of the authors with conversational agents that support health care professionals and young adolescent patients [67,68].

The results of this first feasibility study indicate an overall positive evaluation with respect to the reach of the intervention (ie, 49.5% of 99 young patients approached did install the app and started to interact with the MAX conversational agent), the strong working alliance between patients and the MAX conversational agent, and high acceptance of the intervention by all relevant stakeholders (ie, health care professionals, young patients, and their supporting family members). Compared to very similar conversational agent research targeting childhood obesity [68], physical inactivity [77], or the management of chronic pain [17], this intervention resulted in a high overall therapeutic goal achievement rate (75.5%) but also in improved asthma knowledge test scores and behavioral skills (ie, no identified inhalation mistakes occurred after the feedback from health care professionals). Moreover, the MAX conversational agent was able to motivate family members to support the young patients most of the time when asked (97.8%). In terms of human effort and responsiveness of health care professionals, it can be concluded that the MAX intervention is scalable since most of the conversational turns (99.5%) involved the patients and the MAX conversational agent. After the app onboarding process, which takes an average of 15 minutes, health care professionals had, on average, only one conversational turn with the patients via the manual chat channel of the MAX app when they provided their personalized feedback regarding the inhalation technique. In addition, it took them less than 4 minutes to assess the inhalation technique and 3 days to deliver that feedback to the patients. For each patient, this intervention involved an average of 20 minutes of human effort, 10 automated SMS text messages, including three reminders, and additional costs for gift vouchers, including lottery winnings. We minimized the risk for smartphone addiction [76] by limiting the amount of possible sessions to one per day and further including active exercises outside the digital environment of the app to increase social interaction and to counteract increasing smartphone usage among children [103].

The qualitative feedback suggested several valued and important features, as well as challenges and potential improvements of the intervention. Combining results from each question of the quantitative analysis, and considering the importance and frequency mentioned, several aspects must be discussed and eventually improved in future versions. First, technical issues should be limited as the reach and effectiveness of such an intervention is dependent on problem-free operation. This requires, based on the experience gathered with the MAX intervention, a better understanding and analysis of the technical infrastructure of the health care professionals’ institutions (eg, simple-to-use patient access to broadband internet via Wi-Fi in hospitals). Even though the text-based conversational agent was perceived as positive and engaging, participants indicated that the conversational agent had too many predefined answer options. It was previously suggested that conversational agents can be influential and engaging for young patients, and that open-text answers are highly appreciated [104]. However, privacy issues with conversational agents and open-text answers were pointed out by prior work [105], as conversational agents that are responsive to such inputs could potentially and unintentionally retrieve more and more personal information.

### Limitations and Future Work

This study was designed as a feasibility study with a limited number of participants. It therefore provides the basis, and not the end solution, for future activities in the field. Based on our limited sample, it is clear that the results are not representative and must be interpreted with caution. Further, only health care professionals from four hospitals (eg, pediatric pneumologists) and two cantonal patient organizations of the Swiss Lung Association participated in this study. Therefore, it is not clear whether and to what degree the MAX intervention would work the same way with other relevant health care professionals such as a general practitioner. These nonspecialized health care experts may require significantly more time for the assessment of the inhalation video clips or would not have the expertise to do so without additional educational efforts. Another limitation of this study pertains to the inductive open coding of the interviews that was performed by one author only (SH), resulting in a certain bias of the qualitative results. In addition, since the social support assessment was self-reported by the young patients and linked to additional points for the MAX intervention (to increase the chance to be among the winners), it can be assumed that the supportive involvement of family members was overestimated. Furthermore, the web-based MAX interface for health care professionals (and with it the patient data) was not integrated into hospital information systems or the information system of the patient organization. Specifically, some data had to be stored in a redundant way (eg, contact number, patient name) in the MAX system, which likely resulted in an overestimation of efforts (eg, the duration of the onboarding process). Finally, we have only reported the costs and efforts related to the MAX intervention, and therefore no implications regarding cost-effectiveness can be discussed. It is therefore important that future work investigates the costs of asthma management (eg, the frequency and costs of hospitalizations due to asthma exacerbations) and to which degree they can be reduced with the MAX intervention.

The MAX intervention itself can be improved in several ways. First and foremost, as a next step according to the multiphase optimization strategy [57], we suggest performing optimization trials to identify intervention components that have a positive and significant impact on cognitive and behavioral skills. Toward this end, we suggest assessing the components that are more costly (ie, intervention components that involve human effort). The resulting “effective” intervention package should then be assessed in a final randomized controlled trial with relevant distal health outcomes such as asthma control or quality of life. Moreover, we suggest incorporating a digital biomarker that is able to predict life-threatening events (eg, asthma attacks). For example, there is evidence that the number of nocturnal cough events is negatively correlated with asthma control [106,107], and that nocturnal cough in adult asthma patients can be detected reliably with the microphone of a smartphone [89,108]. Having such a digital biomarker may also help to further develop the MAX intervention as a just-in-time adaptive intervention (JITAI) [109,110]. In such an intervention, after the basic psychoeducational coaching sessions are finished, the MAX conversational agent would message patients only when a specific state of vulnerability [89] and state of receptivity [111] are identified. In addition, and consistent with the JITAI approach, one may also consider an intervention component that monitors medication intake and sends out medication reminders in case no inhalation events were detected. The systematic assessment of inhalation video clips by health care professionals can also be used as a label for the correct use of inhalation devices. Additionally, taking advantage of these labels and the latest advances in video classification methods for activity detection [112] may enable the automatic assessment of inhalation technology. As a consequence, this may reduce the time required to assess the inhalation technique, and may even increase the quality of the assessments. Furthermore, since there was a clear difference in the assessment time of the inhalation video clips between the primary and secondary care settings, a dedicated and specialized expert may be considered for this task. However, this addition may undermine the working alliance between the patient and the primary point of contact (ie, the health care professional who takes care of that patient). Finally, future deployments of MAX must consider a robust, technical infrastructure with a clear focus on the easiest Wi-Fi access possible during on-site consultations to guarantee an efficient download of the app and onboarding process.

In case none of these additional intervention components or studies is considered, estimates of the MAX project team indicate that the development of the current MAX intervention into a “product” would cost another US $100,000. General ongoing costs include keeping the intervention content updated according to recent asthma management guidelines (at a cost of approximately US $10,000 every 3 years) and maintaining technical software (at a cost of approximately US $10,000 per year). In addition, an appropriate legal framework and incentive mechanism has to be established in the Swiss health care system that allows prescription of this “digital pill” by health care providers so that the human and technical efforts, as well as the incentives for the participants (eg, rewards based on the number of intervention points they achieve), can be covered by corresponding payers (eg, individuals or health insurance companies). The recently implemented Digital Healthcare Act in Germany can serve as an example in this regard.

### Comparison With Prior Work

Digital health interventions for asthma include numerous mobile health apps that provide patients with information and help them track symptoms or medications, often using a gamification component [45-47]. A systematic review of 15 different digital interventions for pediatric asthma management showed that 87% of the interventions improved medication and behavioral adherence, and 53% demonstrated improved health outcomes [113]. Although these mobile health apps offer a range of features (eg, automated personalized texts, interactive websites, and online modules) to inform patients about asthma, they have not included scalable text-based health care conversational agents to support communication with health care professionals. Previous studies in other health domains have demonstrated promising results in using conversational agents to improve outcomes, such as promoting physical activity for childhood obesity [104,114]. By applying a scalable conversational agent for asthma specifically, the MAX intervention can provide greater health care professional interaction at reduced cost, which has been a key concern in past asthma interventions [113]. A unique advantage of MAX is its use of a three-component intervention that involves health care professionals, the digital assistant MAX, and family members to support young patients as they work on specific tasks to expand asthma knowledge and improve behavioral skills.

### Conclusions

We have shown that conversational agents framed as digital assistants of health care professionals have the potential to improve cognitive and behavioral skills in chronic disease management, with asthma in children as one specific example. We have demonstrated that conversational agents can take over the role of a mediating social actor in a complex health care setting with various stakeholders, and deliver a digital health intervention in a scalable way into the everyday life of patients and their family members. Consistent with the novel JITAI approach, this study provides further insights into the use of conversational agents that, in the future, may “listen into” states of vulnerability and states of receptivity and, as a result, direct relevant information to appropriate individuals, including the patient, a romantic partner, family member, a nurse, or medical doctor. We therefore envision a future in which scalable conversational agents act like a grand maestro, who dynamically directs an orchestra through a symphony of life based on what the various musicians offer and he or she perceives, and, with each repetition, gets better and better in doing so.

## Appendix

Multimedia Appendix 1 Overview of intervention coaching sessions and schedule (long).

Multimedia Appendix 2 Overview of intervention coaching sessions and schedule (short).

Multimedia Appendix 3 Web-based MAX interface for health care professionals.

Multimedia Appendix 4 Physical onboarding card.

Multimedia Appendix 5 Screenshots of the MAX app.

Multimedia Appendix 6 Video Onboarding, quiz, in-app video.

Multimedia Appendix 7 Video clip of patient inhalation (German).

Multimedia Appendix 8 Screenplay MobileCoach Asthma (German).

Multimedia Appendix 9 Screenplay MobileCoach Asthma (English).

Multimedia Appendix 10 Study recruitment assessment (German).

Multimedia Appendix 11 Study recruitment assessment (English).

Multimedia Appendix 12 Original study flyer (German).

Multimedia Appendix 13 Study flyer (English).

Multimedia Appendix 14 Study information for health care professionals (German).

Multimedia Appendix 15 Video clip: explanation of inhalation assessment.

Multimedia Appendix 16 Study information for patients and family members (German).

Multimedia Appendix 17 Study consent for patients and family members (German).

Multimedia Appendix 18 Survey instruments.

Multimedia Appendix 19 Health Literacy quiz items (English).

Multimedia Appendix 20 Qualitative feedback.

## Abbreviations

BCT: behavioral change technique
CDHI: Centre for Digital Health Interventions
JITAI: just-in-time adaptive intervention
