# Supplementary material for: Conversational Agents as Mediating Social Actors in Chronic Disease Management Involving Health Care Professionals, Patients, and Family Members: Multisite Single-Arm Feasibility Study
Source: J Med Internet Res. 2021 Feb 17;23(2):e25060. doi: 10.2196/25060 (PMC7929753; doi:10.2196/25060)
Supplement: Multimedia Appendix 18 [file jmir_v23i2e25060_app18.pdf]

Survey Instruments

Construct	Subconstruct	Items	Scale / Rating	Numeric Mapping
**Session Assessment**	(A) Perceived usefulness<br>(B) Perceived enjoyment	(A) Did you learn something new?<br>(B) Did you enjoy today's lesson?	**(A)** Tick boxes<br>**(B)** 5-point Likert scale	(A)<br>(1) No, I knew everything<br>(2) Yes, some new aspects<br>(3) Yes, a lot of new aspects<br>(B)<br>(1) Strongly disagree<br>(2) Disagree<br>(3) Hard to say<br>(4) Agree<br>(5) Strongly agree
**Family support**		Have you been supported by your family member?	Tick boxes	(1) Yes<br>(2) Unfortunately not
**Session Alliance Inventory**		1. Max and I respect each other.<br>2. I feel that Max appreciates me.<br>3. I feel that Max cares about me even when I do things that he or she does not approve of.<br>4. Max and I are working toward mutually agreed upon goals.<br>5. Max and I agree on what is important for me to work on.<br>6. I believe the way we are working with my problem is correct.	7-point Likert scale	(1) Never<br>(2) Rarely<br>(3) Occasionally<br>(4) Sometimes<br>(5) Often<br>(6) Very often<br>(7) Always
**Technology Acceptance**	Perceived enjoyment	"I found the app enjoyable"	7-point Likert scale	(1) Strongly disagree<br>(2) Disagree<br>(3) Slighlty disagree<br>(4) Neither<br>(5) Slightly agree<br>(6) Agree
	Perceived ease of use	"The app was easy to use"		
	Perceived control	"I could control many aspects of the app"		
	Perceived usefulness	"The app helped me to increase my knowledge about my asthma"		
	Intention to continue working with the CA	"How much would you like to continue working with MAX?"		
**General Assessment of inhalation video clips**<br>*Note: _ indicates the name of the patient during the assessments*		1. Has _ the correct posture, i.e. an upright upper body, during inhalation?<br>2. Did _ load / prepare the device correctly?<br>3. Did _ exhale enough before inhalation?<br>4. Did _ inhale deeply and long enough through the mouth during inhalation?<br>5. Did _ hold his breath for 5-10 seconds? / Were 10 calm breaths taken via the upstream chamber?<br>6. Did _ exhale slowly afterwards?	Tick boxes	(1) Correct<br>(2) Not correct<br>(3) Not visible in the video clip
**Assessment of inhalation video clips with a focus on metered-doses inhaler, dry powder inhaler, and inhalant containing cortisol**<br>*Note: _ indicates the name of the patient during the assessments*		1. Has the cap of the dosing aerosol been removed?<br>2. Was the metered dose aerosol shaken before inhalation?<br>3. Was the upstream chamber used?<br>4. Was the upstream chamber clean?<br>5. Was the age-appropriate upstream chamber used?<br>6. Was there a whistling sound of the upstream chamber during inhalation? (inhaled too strongly and quickly)<br>7. Did _ trigger the device at the right time during inhalation?<br>8. Was exhaled incorrectly into the powder inhaler so that there is a risk of clumping?<br>9. Has _ rinsed his mouth with water after inhalation or eaten anything?	Tick boxes	(1) Correct<br>(2) Not correct<br>(3) Not visible in the video clip
**Qualitative Feedback**		1. What did you really like about the intervention?<br>2. What needs to be improved in future versions of the intervention?<br>3. What are further suggestions and development options for this intervention?	Open text	NA
