# Supplementary material for: Conversational Agents as Mediating Social Actors in Chronic Disease Management Involving Health Care Professionals, Patients, and Family Members: Multisite Single-Arm Feasibility Study
Source: J Med Internet Res. 2021 Feb 17;23(2):e25060. doi: 10.2196/25060 (PMC7929753; doi:10.2196/25060)
Supplement: Multimedia Appendix 19 [file jmir_v23i2e25060_app19.pdf]

Health literacy quiz items (Schnyder, M.-A. and Santochi, R. Auf Spurensuche im Ferienlager: Ein Lerncomic über Asthma. Lungenliga'.2013.)

Translated by authors, correct answers in **bold**

1. Which statement is false? In asthma, the airways are...

Narrowed

Inflamed

Extended

2. How do you react during an asthma attack?

Inhale, drink something and if possible tell no one

Take a breath-facilitating position, inhale and inform adults

Inhale, hide in a corner, cough as quietly as possible

3. How do the airway-opening emergency medications work?

Fast

Slow

4. How do the anti-inflammatory drugs work?

Short-term

Healing

5. What medication is used in an emergency?

Airway opening medication

Anti-inflammatory medication

6. What can be done about asthma?

Nothing

Knowing and avoiding triggers

Go on holiday in the country

7. What claim about asthma is false:

Asthma is a disease of the small airways (bronchi)

Asthma is contagious

The body lacks oxygen when the airways are severely constricted

8. What is not a sign of asthma?

Palpitations

Whistling noise when breathing

Increased thirst

9. What can trigger asthma symptoms?

Strong smells of paint and food

Loud music

Sweets

10. What is not an allergen?

Animal hair

Mites

Eggs

Oxygen

11. Inflammation is...

... a natural defensive reaction that can occur in all of us

… a pathological reaction in a certain group of people
