# Supplementary material for: Conversational Agents as Mediating Social Actors in Chronic Disease Management Involving Health Care Professionals, Patients, and Family Members: Multisite Single-Arm Feasibility Study
Source: J Med Internet Res. 2021 Feb 17;23(2):e25060. doi: 10.2196/25060 (PMC7929753; doi:10.2196/25060)
Supplement: Multimedia Appendix 20 [file jmir_v23i2e25060_app20.pdf]

Qualitative results from the healthcare proessionals, the patients and the family to question 1 and a: What did you really like about the intervention? (Was war besonders gut an diesem Programm?)									
The following table shows an overview of all identified aspects for the question "What was good about the intervention?". The aspects are sorted according to the frequency of first #HP, then #P and finally #FM.<br>(Note #HP = amount of times the aspect was mentioned by healthcare professionals, #P = by patients, #FM = by family member and #T = amount of times the aspect was mentioned in total)									
**#**	**Coding Term**	**Description**	**#HP**	**#P**	**#FM**	**#T**	**Quote from the involved parties**		
1	Perceived ease of use	The participants and health professionals believed that the application was user-friendly and easy to use.	9	0	0	9	Sie (die Applikation) ist sehr einfach zu bedienen und gut verständlich.”<br>„It (the application) is very easy to use and easy to understand.”<br>MD1		
2	Support from family members	It is a positive aspect that a family member is included in the intervention. The patients should be supported by handling their disease and, therefore, it is good that someone is informed about the intervention.	8	1	1	10	„Ich denke jedoch, dass es wichtig ist, dass die Eltern involviert sind, gerade auch weil die Kinder das Internet benutzen. Somit können sie mitverfolgen, was ihre Kinder genau machen.”<br>„However, I think it is important that parents are involved, especially because children use the internet. Therefore, they can see exactly what their children are doing online.”<br>MD1		
3	Appropriate medium for the target group	The medium is appropriate for the intended target group. It is a medium that the target group accepts, appreciates and likes to use. The patients' willingness to use an application is higher than their willingness to use an offline tool.	7	0	1	8	„Die Kinder sind alle mit dem Handy unterwegs. Ich bezweifle, dass ich Asthma Patienten im jugendlichen Alter für eine andere Asthmaschulung motivieren könnte. Das finden sie halt noch relativ cool, es entspricht genau dem Zeitgeist.”<br>„The kids all carry their smartphones where ever they go. I doubt that I could motivate asthma patients at a young age for a different asthma training. They still think it's relatively cool, it's exactly the spirit of the times.”<br>MD1		
4	Evaluation of the inhalation	The inhaler-technique videos, as well as their evaluation, are regarded as a great advantage of the intervention.	7	0	0	7	„Ich habe dort auch noch einige Fehler gesehen, die mir so, auch beim Demonstrieren oder beim vorzeitigen Nachmachen, nicht aufgefallen sind und das ist natürlich sehr hilfreich.”<br>„I also saw some mistakes (in the inhalation videos) that I didn't notice, even when demonstrating or imitating, and that is of course very helpful.”<br>MD1		
5	Component of asthma counseling	Important that there is an asthma intervention as component of asthma counseling, but it should not replace the personal consultation.	6	0	0	6	Ich finde es sehr gut, dass es wie ein Teil ist von der Asthmaberatung und es sollte auch nur ein Teil bleiben. Es sollte mehr unterstützend sein und nicht etwas ersetzen.”<br>„I think it is very good that the (intervention) is a part of the asthma counseling and it should also stay just a part. It should be mostly supportive and not replacing.”<br>AE3		
6	Expert Cockpit	The Expert Cockpit was a great tool to access the patient's information and progress in the intervention. It was easy to use.	6	0	0	6	„Ja es (Expert Cockpit) war gut. Und auch, dass wir dort schauen konnten, wo sie (die Patienten) stehen.” „Yes, the (Expert Cockpit) was good. And further that we could see where they (the patients) were standing.” AE2		
7	Time period	The time period of the intervention was appropriate.	5	0	1	6	„Und es ist auch nicht zu lang. Es ist nicht so lang, dass es ihnen verleidet. Das fand ich echt gut.”<br>„And it's not too long either. It is not too long that you could not put off with it anymore. I really liked that.”<br>AE2		
8	Future-oriented	This kind of intervention is a forerunner and a right step for the future of health care.	5	0	0	5	„Ich denke, das ist die Zukunft“<br>„I think that this is the future.”<br>AE2		
9	Aesthetics	The aesthetics of the application is age-appropriate. The interface of the application applies design elements, colors and fonts in a proper way and is appealing to the target group.	5	1	2	8	„Ja, ich fand das Design schön und dem Alter angepasst. Der Hintergrund hat mir auch sehr gut gefallen.”<br>„Yes, I found the design beautiful and age-appropriate. I also liked the background very much.” AE1		
10	Standardized evaluation questionnaire	The standardized questionnaire for the evaluation of the inhalation videos was perceived as helpful, practical and time-saving.	5	0	0	5	„Ich fand es sehr gut, dass es standardisierte Sätze gab. Dann vergisst man nichts bei der Beurteilung und muss nicht noch ewig überlegen, wie man das schreiben soll. Das war super.”<br>„I thought it was very good that there were standardized sentences. In this case, you don't forget anything during the evaluation, and you don't have to worry about how to write it. That was great.”<br>AE1		
11	Text-based healthcare conversational agent	The conversational agent (CA) is a great tool and creates great interaction with the patients. The patients perceived the CA as motivating and engaging and liked the design of it. In addition, the patients liked the cooperation with the CA (MAX) and thought that MAX was nice and friendly.	5	12	2	19	„Ich denke, dass mit dem Chat-System ist super. Das geht gut und es gibt eine gewisse Interaktion zwischen uns und dem Teilnehmer über das Tool.”<br>„I think the chat system is great. It works well and provides some interaction between us and the participant.”<br>AE3		
12	Experiential value	The application has a motivating and positive character and was fun to use for the patients. The patients experienced enjoyment and were engaged.	4	2	8	14	„Ich habe manchmal das Leuchten in den Augen der Kinder gesehen. Die hatten Lust, die wollen das. Das ist ein Motivator.”<br>„I've sometimes seen the shining eyes of the children. They wanted to do that. That's a motivator.”<br>MD2<br>„Der spielerische Aspekt punktet voll.“<br>„The playful aspect scores highly. “<br>SFM16		
13	Personal distribution of the application	It is important and beneficial that the application is distributed directly and personally by the health professionals. This way of distribution builds trust, which is extremely important for this kind of intervention.	4	0	0	4	„Die Meinung des Arztes zählt meistens und wenn der Arzt sagt: „Ich denke, das wäre gut für dich", dann wird das auch eher umgesetzt.”<br>„The doctor's opinion counts most, and if the doctor says "I think it's good for you", it's more likely that the patients implement the intervention.”<br>MD4		
14	Acquisition process	The acquisition process was professional and efficient.	4	0	0	4	„Ich glaube nicht, dass es da eine bessere Option gibt. Es ist sehr einfach; man muss nur die Visitenkarte abgeben und damit ist alles klar.”<br>„I don't think there's a better option. It's very simple; you just have to hand them the card and that's it.”<br>MD4		
15	Interaction with the patients	The contact and the interaction with the participants were perceived as helpful and pleasant.	2	0	0	2	„Die positiven Erfahrungen waren der Kontakt mit den Kindern. Das finde ich sehr toll.”<br>„The positive experience was the contact with the children. I think that's very great.”<br>AE1		
16	QR-Code	The QR-code was an effective aid that simplified the installation of the application. It worked well.	2	0	0	2	„Also bei uns war das gut, auch mit dem QR-Code. Ich fand es gut mit dieser Karte und mit diesem Pin (oder QR Code), dass sie (die Patienten) das App direkt finden können und man das ihnen abgeben konnte.”<br>„For us, it was good, also with the QR-code. I thought it was good that with this card and this pin (or QR-code), they (the patients) could directly find the app. We could just give it (the card) to them.”<br>AE5		
17	Personalization	The intervention is personalized since the intervention adapts to the individuals as the patients are called by their name and can choose their own coach (male or female).	2	0	0	2	„Und auch das mit dem Namen geben, finde ich gut, das gibt etwas Persönliches, es ist nicht einfach irgendein System, das mit einem redet. Dann machen die Kinder dann auch lieber.”<br>„Further, giving it a name is good and gives it a personal touch. It's not just a system that you're talking to. The children prefer doing it like it is now.”<br>AE3		
18	Push notifications	Perceived as good; the patients were reminded over push notifications.	2	0	0	2	„Ich fand auch gut, dass es immer wieder ein Reminder gegeben hat.”<br>„I also liked the fact that there was always a reminder.”<br>MD1		
19	Learning videos	The learning videos were perceived as great and helpful.	1	5	0	6	„Die Fime sind super.”<br>„The movies are great.”<br>MD3		
20	Quiz	The quiz at the beginning and end was a great tool for patients to track their progress and see their results.	1	3	0	4	„Und was ich auch sehr gut fand, war das Quiz am Anfang und am Schluss, dann haben sie auch gesehen, was können sie besser, was haben sie verstanden und was vielleicht noch nicht.”<br>„And what I also liked very much was the quiz at the beginning and in the end, which made them see what they were able to do better, what they understood and what they maybe didn't understand yet.”<br>AE5		
21	Time flexibility	The patients have the freedom to go through the lessons according to their individual time schedule.	1	0	1	2	„Zudem ist es zeitlich flexibel, also man kann es machen, wenn man Zeit hat.”<br>„It's also flexible in terms of time, so you can do it when you have time.”<br>AE2		
22	Point system	The point system motivated and convinced the participants to take part in the intervention.	1	0	0	1	„Und für die Jugendlichen war das Gewinnspiel sehr überzeugend. Damit konnte ich sie fast immer locken, das war recht wichtig für sie.”<br>„And for the young people, the competition was very convincing. I could almost always lure them with it, it was quite important to them.”<br>MD1		
23	Supervisor chat (in the application)	The supervisor chat was perceived as helpful. The opportunity to chat directly with the healthcare professionals was appreciated.	1	1	0	2	„So können die Kinder auch mit dem Betreuer Probleme besprechen. Das ist sehr gut.”<br>„For instance, the children can also discuss problems with the supervisor. That's very good.” MD2		

24	Educational content	The content of the intervention was perceived as helpful and educational.	0	16	24	40	„Die Information rund um das Thema Asthma waren sehr lehrreich“<br>"The information on asthma was very educational."<br>SFM15		
25	General satisfaction	Everything of the intervention was perceived as good.	0	4	1	5	„Alles👍“<br>„Everything 👍“<br>PAT21		
26	Experiments	The experiments in the intervention were perceived as enlightening and educational.	0	2	3	5	„Die Experimente mit Röhrli und zugeklebter Flasche war für mich als Mutter sehr aufschlussreich!“<br>„The experiment with the straw and the glued bottle was very informative for me as a mother!”<br>SFM18		
27	Children's autonomy	It was appreciated that the children (patients) were able to carry out the intervention almost independently.	0	0	3	3	„Dass es die Kinder fast selbständig machen können.“<br>„That the children can almost do it on their own. “<br>SFM23		

Qualitative results from the healthcare professionals, the patients and the family members to question 2 and b: What were the difficulties and challenges of using the intervention? What needs to be improved in future versions of the intervention? (Was muss unbedingt an diesem Programm verbessert werden?)							
The following table shows an overview of all identified aspects for the question "What needs to be improved in future versions of the intervention?". The aspects are sorted according to the frequency of first #HP, then #P and finally #FM. (Note #HP = amount of times the aspect was mentioned by healthcare professionals, #P = by patients, #FM = by family member and #T = amount of times the aspect was mentioned in total)							
**#**	**Coding Term**	**Description**	**#HP**	**#P**	**#FM**	**#T**	**Quote from the invovled parties**
1	Smartphone availability	Potential participants for the intervention do not necessarily have a smartphone or do not have an internet-enabled smartphone.	9	0	0	9	„Häufig war das Problem, dass bei möglichen Teilnehmern nicht zwei internetfähige Handys vorhanden waren. Das heisst, die Kinder hatten noch kein Handy zur Verfügung."<br>„Frequently, the problem was that potential participants did not have two internet-enabled smart phones. This means that the children did not yet have a smart phone at their disposal."<br>AE1
2	IT problems (Expert Cockpit)	The Expert Cockpit could not be opened as expected or the functions of the cockpit could only be used to a limited extend. Most of these problems were caused by the internet browsers (such as Safari or Google Chrome) and/or hospital computers with limited access to the internet).	6	0	0	6	„Ich konnte anfänglich das Dashboard (Expert Cockpit) nicht benutzen, da die Seite von der IT des Spitals gesperrt wurde."<br>„I could not use the Dashboard (Expert Cockpit) at first, because the site was blocked by the IT of the hospital."<br>MD2
3	Lack of notification (healthcare professionals)	The notifications or feedbacks for the healthcare professionals were insufficient. They did not get informed about the development of their patients via e.g. messages in the supervisor chat. It is important that they receive a notification when their patients make progress, send messages, or stop the intervention.	6	0	0	6	„Ein weiteres Problem ist, dass wir nicht informiert werden, wenn ein Patient die Intervention beendet hat, ein Problem hat oder sein Video nicht hochlädt. So können wir nicht intervenieren und herausfinden, an was es liegt."<br>„Another problem is that we were not informed when a patient had finished the intervention, had a problem or did not upload a video. So, we are not able to intervene and find out what the problem was."<br>AE2
4	Time efforts (Healthcare professionals)	Finding suitable patients, installing the application, and supervising the patients was time-consuming. A certain commitment is required.	6	0	0	6	„Das (Beraten für die App) geht nur vereinzelt, aber wenn ich das dauerhaft mache, habe ich einfach die Zeit nicht. Im Rahmen der Studie ist die Zeit schon nicht zu unterschätzen."<br>„That (consulting for the app) is only possible in certain cases, but if I would do it permanently, I just don't have the time. As part of the study, the time needed is not to be underestimated."<br>MD3
5	Insufficient access to potential participants	The Patient Organization does not have enough children with asthma who fit the criteria of the intervention. The focus of the Patient Organization lies more on other pulmonary diseases or older patients.	5	0	0	5	„Bei uns war es intern ein wenig schwer, weil wir nicht so viele Kinder zur Verfügung hatten, da wir bei uns mehr erwachsene Patienten haben. Zudem bieten wir hier auch nicht direkt Asthmaschulungen für Kinder an."<br>„It was a little difficult for us internally, because we didn't that many children available, as we have more adult patients with us. In addition, we don't offer asthma training directly for children here."<br>AE5
6	Strict inclusion criteria	The (seven) inclusion criteria were too restrictive and only allowed for a small number of potential participants.	5	0	0	5	„(...), es war höchstens so, dass sie (die möglichen Patienten) die Kriterien nicht erfüllten. Die Einschlusskriterien dieser Studie waren sehr streng."<br>„(...), it was at most that (the possible patients) did not meet the criteria. The inclusion criteria for this study were very strict."<br>MD4
7	Study setting	The fact that the intervention was part of a study and various forms (such as the declaration of consent) had to be signed was an obstacle to participate for several potential patients (and their supporting family member).	4	1	0	5	„Das sie nicht begonnen haben, ist sicher wegen der Studie, wegen dieser ganzen Sache mit der Einverständniserklärung und all diesem Zeug. Wahrscheinlich hat das die Eltern abgeschreckt."<br>„They didn't start because of the study, because of this whole process with the consent form and everything. Probably that scared the parents off."<br>AE4
8	Not age-appropriate	The application was too childish and not age-appropriate for the older patients of the intervention.	4	0	1	5	„Vielleicht hätte man die Inhalte noch ein wenig schärfer machen sollen. Also sagen wir es so: Für ein 14-15-Jährigen ist es ein bisschen zu banal. Ich glaube die Instruktionsvideos sind von der Art schon tendenziell für Kinder zwischen 8-13 Jahre."<br>„Perhaps the content should have been made a little spicier. So, let's put it this way: it's a bit too trivial for a 14-15-year-old. I believe the instructional videos (and the app) tend to be for children between 8-13 years old."<br>MD4
9	Compensation (healthcare professionals)	The healthcare professionals need to be compensated for the time invested in the intervention.	3	0	0	3	„Ärzte müssen wissen, dass sie die Abwesenheitsarbeit (in Abwesenheit des Patienten) verrechnen können und so weiter. Ich weiss aber nicht wie das genau aussieht, ob sie das einfach so dürfen. Aber die Ärzte machen es ja nicht kostenlos, daher muss es ihnen auch etwas bringen."<br>„Doctors need to know that they can charge for absent work (i.e., in the absence of the patient). But I don't know if they are allowed to do so. But the doctors don't work for free, so it has to be advantageous for them."<br>AE4
10	Privacy concerns	Concerns were brought up from the patients or the family member regarding privacy issues and data protection.	3	0	0	3	„Ich habe allen gesagt, dass alles verschlüsselt ist und dass das keiner sehen wird. Sie hatten dann doch Bedenken, dass das irgendwo im Internet rumkursiert."<br>„I told everyone that everything was encrypted, and no one would see it. But they were still worried that it could circulate somewhere on the internet."<br>AE3
11	Instructions about inhalation video clips	Instructions about the exercise of the inhalation video clip should contain more detailed information, e.g. via the chat, to ensure that patients do the exercise correctly and record the inhalation properly.	3	1	0	4	„Wichtig ist es, besser zu betonen, was das Video alles enthalten sollte. Es ist vorgekommen, dass die Patienten das Video unvollständig gemacht haben."<br>„It is important to better emphasize what the video clip should contain. It happened that patients produced an incomplete video."<br>MD2
12	Acquisitions problems	There were difficulties with the acquisition process. For example, some potential participants did not respond, some did not answer and some never sent the forms back. This was time-intensive.	3	0	0	3	„Also, ich fand das Akquirieren nicht ganz so einfach. Zum Teil hat man sie (die möglichen Teilnehmer) nicht erreicht und zum Teil haben sie gesagt, dass sie mitmachen, haben dann aber doch nicht mitgemacht."<br>„Well, I didn't find the acquistion process very easy. Partly, they (the possible participants) could not be reached and partly they said that they would participate, but then they did not start the intervention."<br>AE2
13	Recruiting location	The difficulty is where to find children with asthma who are suitable for this intervention.	3	0	0	3	„Von dem her, ich weiss gar nicht, ob diese Asthmakinder überhaupt in der Lungenpraxis bekannt sind oder ob die einfach vom Hausarzt oder vom Kinderarzt betreut werden."<br>„From that point of view, I don't even know if children with asthma are known in lung practices or if they are simply supported by their general practitioner or pediatrician."<br>AE1
14	Lack of support from family member	Either there is a lack of a suitable family member or the family member does not have enough time or resources to properly support the patient.	3	0	0	3	„Denn wenn die Eltern nicht mitmachen wollen, haben die Kinder die Chance gar nicht, mitmachen zu können. Und das finde ich wiederum sehr schade."<br>„If the parents are not willing to participate, the children don't even have a chance to participate. That's a pity."<br>AE3
15	Ending	The ending of the intervention was not properly communicated with e.g. an end message from the healthcare professionals or an overall feedback from the patient to the healthcare professional.	3	0	0	3	„Ja, es wäre schön, wenn von uns dort am Ende noch eine Rückmeldung kommen würde wie z.B. "Toll hast du mitgemacht. Die Intervention ist jetzt beendet."<br>„Yes, it would be nice if there would be some feedback from us at the end like "You did a great job. The intervention is now finished." AE2
16	Intervention guidelines	Healthcare professionals perceived uncertainty on how to proceed when a patient stopped the intervention. Instructions or guidelines on how to intervene were missing.	2	0	0	2	„Wenn ich sehe, dass jemand nicht weiter macht, wie soll ich dann vorgehen? Mal habe ich geschrieben, mal habe ich angerufen. Da wäre eine Anleitung gut."<br>"When I see someone stopping (the intervention), how am I supposed to proceed? Sometimes I wrote, sometimes I called them. An instruction would be good."<br>AE2
17	Ambiguities with the QR- code	Patients were insecure with the QR-code or could not scan it directly (android phones).	2	0	0	2	„Was manchmal noch das Problem war, dass einige nicht viel mit dem QR-Code anfangen konnten. Also dort war das Scannen des QR -Code ein Hindernis."<br>„It was sometimes the problem that some people couldn't manage the QR-code. In this cases, scanning the QR-code was an obstacle."<br>MD3
18	Point system	The point system was perceived as unnecessary and childish.	2	0	0	2	„Ja also ich fand das mit den Punkten ein wenig blöd. Also ich hätte jetzt diese Punkte nicht gebraucht."<br>"Yeah well, I thought it was a little stupid with the points since I didn't really need those points."<br>AE2
19	Healthcare professional support	The healthcare professionals need support in the acquisition and supervision of the intervention. (Studynurse)	2	0	0	2	„Die haben einfach nicht angefangen. Dies ist natürlich in meinem Fall schlecht. Ich habe dann nicht die Zeit, da hinterher zu telefonieren. Es wäre da gut, wenn es da eine Studynurse gibt, die da telefonieren könnte."<br>„They (the patients) just didn't start it. This of course is bad. For this reason, I don't have the time to call afterwards. It would be good if there was a study nurse that could make the phone calls."<br>MD3
20	Too much predefined text	The conversational agent only has a few answer options and too many suggested answer possibilities. More answer possibilities or free text options would improve interaction.	2	10	9	21	„Ich denke das einzige was ein wenig fehlt ist, dass man ab und zu selbst etwas schreiben kann. Man muss wirklich von Anfang an Antworten anklicken und dadurch fühlt es sich schon nicht ganz so natürlich an."<br>„I think the only thing missing a little is that you can't write anything yourself now and then. You really have to click on answers starting from the beginning and this doesn't feel very natural."<br>AE4<br>„Man soll die eigene Meinung einbringen können und die Antworten sollen nicht vorgegeben sein."<br>"One should be able to contribute one's own opinion and the answers should not be predefined."<br>PAT29
21	Long-winded	The onboarding process, as well as some parts during the intervention, are too long-winded.	2	0	2	4	„Bis es mal tatsächlich irgendetwas mit Asthma zu tun hat geht es schon lange."<br>„It takes a long time until it really deals with asthma."<br>AE4

22	Internal WIFI problems	The internal WIFI of the institutions was not working for the patients and they could not download the application.	2	0	0	2	„Für mich persönlich wäre am wichtigsten, dass das Internet hier im Spital funktioniert. Dass die Kinder sich mit dem Gästeaccount anmelden können und ich es gemeinsam mit ihnen installieren kann." „For me personally, the most important thing would be that the internet works here in the hospital. That way, the children could log in with a guest account and I could install it together with them." MD1
23	IT literacy	The healthcare professionals need to have certain knowledge and ability to utilize the application and use/handle the dashboard efficiently and in order to explain it to potential patients.	2	0	0	2	„Ich habe bemerkt, dass meine Kollegen, denen ich es erklärt habe, Bedenken hatten, dass sie es den Patienten nicht vernünftig erklären könnten." „I have noticed that my colleagues to whom I have explained it had concerns that they could not reasonably explain it to patients." MD1
24	Smartphone usage	The smartphone usage of the patient is a critical topic. The usage per day should be not too long and it should be limited.	2	0	0	2	„Ich konnte sagen, die Nutzung ist beschränkt auf eine Viertelstunde pro Tag und nur für zwei Wochen. Sonst kommen da schon noch deutlich mehr Bedenken auf." "I could say that the usage is limited to a quarter of an hour per day and only for two weeks. Otherwise, there would be more concerns." MD1
25	Too many superlatives	The messages have a character which suggests a positive and optimistic answer (often with superlatives). The patient does not have the option to choose something negative.	1	0	0	1	„Und es gibt nur Antwortmöglichkeiten, die positiv formuliert sind und keine Aussagen, dass man es schlecht findet. Und dann muss man halt solche superlativen Ausdrücke anwählen, obwohl man das nicht so fühlt. Wenn man etwas anderes angeben muss als das, was man fühlt, dann gibt das eine kognitive Dissonanz. Dies ist langfristig nicht erfolgsversprechend." „And there are only answer possibilities that are formulated positively and none that say that you don't like anything. And then you have to choose such superlative expressions, although you don't feel that way. If you have to say something other than what you feel, it results in cognitive dissonance. This is not promising in the long run." AE4
26	Restricted amount of session per day	Patients can only do one session per day. The option to do more on (for example on the weekend) does not exist.	1	0	0	1	„Ich konnte nicht vorwärts gehen, da Fragen und Antworten begrenzt waren und erst am nächsten Tag weitergingen." MD2 „I couldn't go forward because questions and answers were limited and only continued on the next day." MD2
27	Inhalation types	There are only three possible inhalation types presented in the intervention. There are probably more types. Leave a blank space for other options.	1	0	0	1	„Ich habe mich aus ärztlicher Sicht noch gefragt, ob es wirklich nur diese drei Arten der Inhalation gibt. Es wurden einfach die drei Häufigsten aufgelistet und ich weiss nicht ob es da vielleicht noch mehr oder neue gibt." „I asked myself from a medical point of view whether there are really only these 3 types of inhalation. The three most common ones were simply listed and I don't know if there are more or new ones." AE4
28	Downloading problems	The patient was unable to download the application with his/her smartphone (Google phone).	1	0	0	1	„Das Kind hatte ein Google Handy, und dort konnte man es dann nicht herunterladen. Aber ich glaube da war das Handy das Problem. Ich glaube das Handy war nicht kompatibel." „The kid had a Google smartphone and you couldn't download it there. But I think the problem was the smartphone. I don't think the phone was compatible." AE5
29	Accessibility (Expert Cockpit)	Accessing the cockpit was tedious.	1	0	0	1	„Gut vielleicht, den Zugang noch ein wenig vereinfachen, also dort wo man die Inhalationsvideos beurteilt. Dass man dort noch schneller reinkommt." „Well maybe, simplify the access to where we judge the inhalation videos a bit (i.e., the cockpit) so that you can log in there even faster." AE5
30	Limited asthma personalization	Some patients do not have to inhale regularly. They cannot record the inhalation video at any time. There should be an option to postpone the recording. Other patients do not have asthma attacks or do not inhale at all, for these some lessons are redundant. There should be a way to indicate these individual requirements in order to personalize the intervention accordingly.	1	2	1	4	„Es gib viele die inhalieren gar nicht täglich und die können dann nicht sofort ein Video aufnehmen. Nur die starken Asthmatiker nehmen sicher jeden Tag ein Medikament zu sich." „There are many who don't inhale daily and they can't record a video immediately. Only the strong asthmatics have to take a medicine every day." AE4
31	General satisfaction	No suggestions for the improvement of future interventions were mentioned by the involved parties.	0	18	11	29	„Ich persönlich finde sie gut so wie sie ist. " „Personally, I think it's good the way it is. " SFM21
32	Lack of deleting options	The option to delete answers was missing.	0	2	1	3	„Das man antworten wieder löschen kann." „That you can delete answers." PAT20
33	Limited understandability	The understandability of the app was limited as some aspects were unclear to the participants and their supporting family member.	0	2	1	3	„Die Ziele besser festlegen." „Define the goals in a better way." PAT37
34	Technical issues with responses from MAX	A patient had technical issues with the application and the conversational agent responses and the messages from the CA MAX were delayed.	0	0	1	1	„Die Antworten von Max liessen zwischendurch unnatürlich lange auf sich warten." „Max's answers were unnaturally delayed from time to time. " SFM27
35	Perceived shared control	The tasks with the family member could be started without an official conformation of the family member. The family member suggested a shared decision making for the tasks where the patient and the family member work together.	0	0	1	1	„Wenn das Kind Unterstützung von der Helferperson für die Tagesaufgabe benötigt, hätte ich es für gut befunden, dass erst durch ein Okay der Helferperson die Aufgabe gestartet werden kann. " „If the child needs support from the supporting member for a daily task, I would have preferred it if the patient could only start the task with an OK of the supporting person." SFM13

Qualitative Results from the healthcare professionals, the patients and the family members to question 3 and b: What are further suggestions and future development options for this intervention? (Was muss unbedingt an diesem Programm verbessert werden?)							
The following table shows an overview of all identified aspects for the question "What are further suggestions and future development options for this intervention?". The aspects are sorted according to the frequency of first #HP, then #P and finally #FM. (Note #HP = amount of times the aspect was mentioned by healthcare professionals, #P = by patients, #FM = by family member and #T = amount of times the aspect was mentioned in total)							
**#**	**Item**	**Description**	**#HP**	**#P**	**#FM**	**#T**	**Quote from the involved parties**
1	Adaption of the inclusion criteria	The inclusion criteria were too strict and should be adapted. Adaptions could be made regarding to age, internet-enabled smartphone and/or supporting family member)	5	0	0	5	„(...), ich habe gerade gedacht, das Alter noch etwas zu erweitern, nach unten zum Beispiel." "(...), I was just thinking of extending the age a little bit, downwards for example." AE1
2	Cooperation with pneumologists and GPs	For the future, it is important that also pneumologists and GPs are informed about the intervention and that patients from these practices get recruited as well.	3	0	0	3	„Um mehr Teilnehmer zu finden, denke ich, müsste man zu den Kinderärzten gehen, also so ein wenig an die Front gehen, wo man die Kinder auch direkt erreicht. Es gibt auch Kinderärzte, die viele Inhaliergeräte abgeben, dort könnte man gut andocken und rekrutieren." „In order to find more participants, I think you would have to go to the pediatricians, to go a little to the front where you can reach the children directly. There are also pediatricians who supply a lot of inhalers, you could easily recruit there." AE5
3	Expansion to other health related topics or diseases	The intervention could be expanded to other chronical diseases or other health related topics (e.g. smoking, eating disorders).	3	0	0	3	„Dies könnte auch für andere chronische Krankheiten verwendet werden, aber es kommt auf die Komplexität der Erkrankung an. Insgesamt ist es sehr ausbaufähig." „This could also be used for other chronic diseases, but it depends on the complexity of the disease. Overall, it is very expandable." MD1
4	Further interaction	Generate more and further interaction between the healthcare professional and the patient during or after the intervention (e.g. responses to all uploads, follow-up questions, re-uploads).	3	0	0	3	„Vielleicht müsste man sich überlegen, ob man im Nachhinein noch etwas machen will. Zum Beispiel, dass man noch mal eine Meldung senden würde oder nachfragen würde wie es mit dem Asthma geht." „Maybe you'd have to consider doing something afterwards. For example, that you would send a message again or ask how they are doing with their asthma." AE2
5	Other reminders	The app should remind the patient to take the medication, inhale etc. These reminders could also be over a longer period than the intervention.	2	0	0	2	„Aber man muss das Potenzial ausschöpfen. Medikamentenerinnerungen müssen da unbedingt rein. Inhalationserinnerung. Auf jeden Fall so eine Erinnerungsfunktion." „But you've got to make the most of it. Reminders about medications must be included. Inhalation reminders. In any case, a reminder function." MD3
6	Examining the physical and mental state of the patient	Examine the physical and mental state of the patient with the application. With that, problems and inconveniences of the treatment could be discovered.	2	0	0	2	„Vielleicht wäre es dann möglich, die Befindlichkeit des Patienten aufzunehmen damit man herausfinden kann, warum er das nicht möchte oder welche Gründe ihn dazu bewegen (die Medikamente nicht zu nehmen). Das wäre noch wichtig für die Therapie-Adhärenz. Es gibt Gründe, die vielleicht lösbar sind (...). Es wäre gut, wenn sie (die Patienten) dies mit dem Asthmacoach besprechen könnten und dass dies dann eben mir zugetragen wird." „Perhaps it would then be possible to record the patient's mental state so that one can find out why he does not want this or what reasons motivate him (not to take the medication). This would also be important for therapy adherence. There are reasons that may be solvable (...). It would be great if they (the patients) could discuss this with the asthma coach and that this is then brought to my attention." MD2
7	Expansion to other target groups	The app should be adapted and expanded to other target groups such as older teenagers or adults.	2	0	0	2	„Ich würde sagen für ältere Patienten könnte man das App noch ausbauen. " „I'd say for older patients, the app could be expanded." AE3
8	Long-term management, therapy and coaching	The application should expanded to an asthma management application. This means that the patients use the application for their long-term therapy and coaching. The application must help the patient to manage his/her disease successfully. The application should further offer support and create a bridge between the medical appointments.	2	0	0	2	„(...) Es wäre dann wichtig, dass die Patienten über das App eine Dauertherapie durchführen könnten. (...) Das App kann zwischen den ärztlichen Kontrollen Kontakt schaffen, erinnern, die Inhalationstechnik mittels Videos überprüfen, und auch die Häufigkeit der Inhalation überwachen." „(...) It would then be important for the patients to be able to carry out permanent therapy via the app. (...) The app can establish contact between the medical controls, remind, check the inhalation technique with videos, and also monitor the frequency of inhalation." MD2
9	Public version	Discussion and ideas about a public version (without for e.g. the QR-code or the inhalation video).	2	0	0	2	„Ich glaube eine Veröffentlichung ist etwas, was durchaus Sinn machen würde. Die Frage ist, wenn man es jetzt öffentlich zugänglich macht und es unkontrolliert veröffentlicht wird, wer begleitet das Medizinische, wer schaut die Videos an?" „I think a public release is something that would make sense. The question is if you make it public now and it is published uncontrollably, who is accompanying the medical aspect, who is watching the videos?" MD4
10	Examining therapy adherence and compliance	Examine the therapy adherence and compliance with the application. This means checking the frequency of the patient's inhalation or/ and medication intake.	2	0	0	2	„Im Ausbau der Therapieüberwachung mit Möglichkeiten zu Rückfragen wie: Wie geht es dir? Hilft das Antiasthmatikum? Nebenwirkungen? Etc. Dazu wäre noch die Aufzeichnung der Compliance, also wie oft jemand inhaliert hat." „In the expansion of therapy monitoring with opportunities to ask questions such as: How are you doing? Does the antiasthmatic help? Side effects? Etc. in addition, there would be the recording of compliance, i.e. how often someone has inhaled." MD2
11	Expand content of the intervention	Expand the content of the intervention to therapeutic procedures, effects of the therapy for the patient and ACTs (asthma control test).	2	0	0	2	„Ich würde vielleicht noch mehr nachfragen über die Medikamente und was für Vorteile das sind und nicht nur über das Asthma und was das genau ist. Man müsste es ausweiten und quasi im Hinblick auf das Verständnis auch das therapeutische Vorgehen und was draus für den Patienten folgt erklären." „I might ask more about the medications and what benefits they bring and not just about asthma and what it exactly is. It would have to be expanded and, in terms of understanding, the therapeutic procedure and what follows for the patient would have to be explained." MD1
12	Collecting inhalation data	Examine the quality of the inhalation of the patients over the data collected in the application.	2	0	0	2	„Denn diese Videos beinhalten Informationen, die extrem spannend sind, wie zum Beispiel wie gut die Patienten die Inhalation machen oder wie gut sie instruiert sind." „Because these videos contain information that is extremely interesting, such as how well the patients do the inhalation or how well they are instructed." MD4
13	Language barriers	Potential patients or their family member do not understand the language. Illiterates cannot use the intervention.	2	0	1	3	„Die Sprache ist auch ein Problem. Bei ausländischen Eltern, die nicht gut Deutsch verstehen, wird es schwierig, da sie meistens viele Bedenken haben, weil sie es wahrscheinlich nicht verstehen." „Language is also a problem. For foreign parents who don't understand German well, it becomes difficult, because they usually have many concerns since they probably don't understand it." AE3
14	Gaming components	It might be an idea to integrate gaming components into the intervention to make it more attractive for the children.	1	0	0	1	„Was ich in einer anderen Studie noch schön fand, war, dass es eine Art Game integriert hatte. (...) Vielleicht, um die Kinder mehr zu faszinieren, wäre das schon noch eine Überlegung." „What I liked about another study was that it had some kind of game integrated into it. (...) Maybe, to fascinate the children more, that would be another consideration." MD3
15	Utilize all the capabilities of a smartphone	Further interventions on applications should utilize all the capabilities of a smartphone.	1	0	0	1	„Die Möglichkeiten, welche die Smartphones in sich tragen, diese müssen in jedem Aspekt genützt werden; Bewegungssensoren, Geräuschsensoren, Interaktion über einen Chat." „The possibilities that smartphones carry must be used in every aspect; motion sensors, sound sensors, interaction via chat." MD4
16	Follow-up intervention	Offer a follow-up application that teaches some topics more in-depth and offers additional information.	1	1	3	5	„Ausbaumöglichkeiten hat man in dem Rahmen, dass man sich auf die Schulung bezieht und noch vertiefter teached. Aber das müssten dann sicher neue Installationen und Sachen sein." „There are expansion possibilities within the framework so that one refers to the first intervention and then teach it even more profoundly. But that would need new applications." MD1
